# Resveratrol-embedded hollow cerium oxide nanomedicine targeted treat inflammatory bowel disease through ROS clearance, intestinal mucosal immune homeostasis recovery and gut microbiota modulation

**DOI:** 10.1016/j.mtbio.2026.102765

**Published:** 2026-01-07

**Authors:** Tianlin Wang, Xiaoxia Lin, Wenjie Li, Xing Li, Xiaodong Lin, Ning Li, Yan Ma, Lianjun Song, Xianqing Huang, Tiange Li

**Affiliations:** a College of Food Science and Technology, Henan Agricultural University, Zhengzhou, 450002, China; b College of Public Health, Zhengzhou University, Zhengzhou, 450000, China; c Department of Bioengineering, University of California, Riverside, CA, 92521, United States

**Keywords:** Inflammatory bowel disease, Resveratrol-embedded hollow cerium oxide, ROS-Inflammation clearance, Intestinal mucosal immune homeostasis recovery, Gut microbiota modulation

## Abstract

Inflammatory bowel disease (IBD) is a chronic inflammatory disorder of the gastrointestinal tract that is difficult to cure. The crucial pathogenic factors of IBD are mainly caused by the overexpression of pro-inflammatory cytokines and the disturbance of gut microbiota triggered by excessive reactive oxygen species (ROS). Herein, the resveratrol-embedded hollow cerium oxide composite nanomaterials with surface modified hyaluronic acid (Res-CeO_2_@HA) is developed to restore intestinal mucosal immune homeostasis and modulate gut microbiota via effective elimination of ROS-inflammation. The synthetic nanomedicine integrates the enzyme-like activity of CeO_2_, the antioxidant properties of Res, and the targeting capabilities of HA. Results showed that Res-CeO_2_@HA had significant advantages in ROS clearance and colon targeting. And it balanced the expression of inflammatory cytokines by inhibiting M1 macrophage polarization, promoting M2 macrophage polarization, and modulating the TLR4/NF-κB signaling pathway to alleviate IBD in mice. Furthermore, it is found that Res-CeO_2_@HA significantly improved the homeostasis of the intestinal microbiota. This friendly and multifunctional nanomedicine may provide new strategies for the clinical treatment of IBD.

## Graphical abstract

**Figure:**
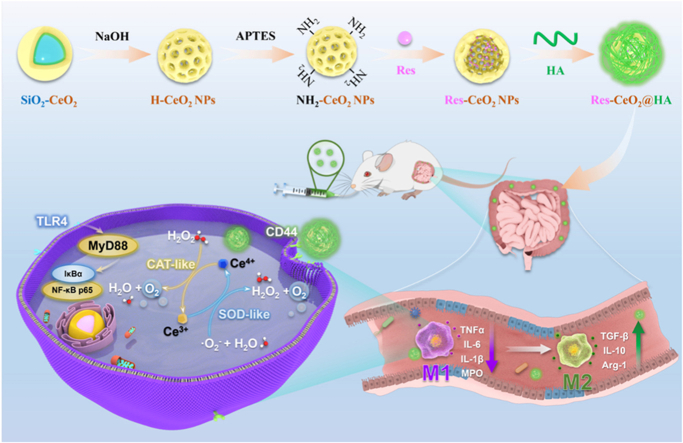

## 1 Introduction

Inflammatory bowel disease (IBD) is a chronic intestinal inflammatory disease that includes two subtypes: ulcerative colitis (UC) and Crohn's disease (CD). It is prone to recurrence and cannot be completely cured. IBD has a significant impact on physical health, as evidenced by its alarming clinical symptoms such as weight loss, abdominal pain, bloody stools, and the potential development of colon cancer [[1], [2], [3]]. The conventional treatment method is to use antibiotics and immunosuppressants for clinical intervention. However, frequent and prolonged use of medication can lead to multiple complications, including autoimmune disorders, liver damage, and malignant tumors [4,5]. Therefore, developing effective and safe IBD treatment strategies is highly anticipated.

Although the pathogenesis of IBD remains unclear, mounting evidence indicates that excessive production of ROS is one of the significant etiological factors in the pathogenesis of IBD. Excessive ROS originating from gastrointestinal mucosal cells can trigger inflammatory responses, resulting in excessive secretion of pro-inflammatory cytokines [6,7]. One of the molecular mechanisms involves the sustained activation of NF-κB, which is induced by excessive ROS. This causes the release of increased cytokines to further activate NF-κB, thus inducing an inflammation cascade and ultimately forming a positive feedback loop regulation mode of “inflammation-NF-κB-inflammation” [8,9]. Furthermore, excessive stimulation of pro-inflammatory cytokines activates immune cells, including macrophages and neutrophils, which is accompanied by impaired intestinal barrier function and symptoms of intestinal microbiota imbalance [10,11]. Therefore, developing promising strategies capable of effectively restoring intestinal mucosal immune homeostasis and regulating intestinal microbiota by eliminating ROS-inflammation responses holds immense practical significance for the clinical treatment of IBD [12,13].

Recently, many treatment strategies have been proposed to address the complex interaction between ROS and inflammatory response. However, many of them often lack specificity, potentially interfering with other normal physiological processes, leading to unwanted side effects [14]. Similarly, anti-inflammatory drugs typically focus on a specific stage of the inflammatory process, potentially failing to comprehensively and effectively suppress the entire process, resulting in limited therapeutic effects [15]. Natural polyphenols, with their diverse biological activities and excellent biological safety, have been regarded as important substances for alleviating inflammatory conditions and other diseases [16]. As one of the polyphenols, Resveratrol (Res) is widely present in plants such as *Polygonum cuspidatum* and grapes [17,18]. Res possesses multiple benefits including anti-inflammatory, antioxidant and immune-regulatory activities which has been widely used in inflammation [19], obesity [20] and cardiovascular diseases [21] treatment. Res could reduce local inflammatory responses and inhibit the polarization of M1 macrophages by clearing excess ROS [22]. More importantly, it also has prominent functions in regulating gut microbiota and improving intestinal barrier dysfunction [23]. However, oral administration of Res is reportedly insufficient to treat IBD, as it is poorly absorbed and extensively metabolized in the gastrointestinal tract [19].

To overcome these challenges, synthetic nanozymes with ROS scavenging ability have been designed as friendly and targeted methods for treating IBD because of their excellent biocompatibility and enzyme-like activity. Among them, the application of hollow cerium oxide (CeO_2_) nanoparticles in the field of nanomedicine has garnered widespread attention based on the reversible conversion between Ce^3+^ and Ce^4+^. The characteristics of enzymatic activity of CeO_2_ are similar to superoxide dismutase (SOD) and catalase (CAT) [24]. Hence, CeO_2_ exhibits great potential among the treatment of diseases intimately associated with excessive production of ROS and inflammatory responses, such as acute lung injury [25], ischemic stroke [26] and rheumatoid arthritis [27]. In addition, studies also have shown that CeO_2_ can suppress polarization of M1 macrophages (secretion of pro-inflammatory factors) while promote polarization of M2 macrophages (secretion of anti-inflammatory factors) [28], and alleviate the local inflammatory response by removing excessive ROS, thus reducing the release of inflammatory cytokines [29]. Therefore, CeO_2_ is was selected as an excellent candidate for treating IBD. However, most nanozymes struggle to achieve comprehensive and efficient clearance of the complex and continuously produced ROS *in vivo* [30], relying solely on its antioxidant ability [24]. In current research on CeO_2_ nanoparticles for the prevention or treatment of IBD, CeO_2_ nanozymes were integrated with various materials (e.g. biohybrid sporopollenin, *Spirulina platensis* or probiotic outer membranes) to develop delivery systems to protect the nanoparticles from degradation in the gastric microenvironment and enable their rapid release in the intestinal tract [[31], [32], [33]]. On the other hand, CeO_2_ nanoparticles can be used as a potential computed tomography (CT) contrast agent for imaging gastrointestinal tract with IBD while protecting against oxidative damage [34]. Nevertheless, few studies have reported the construction of intestinal-targeted nanosystem via loading food-derived bioactive components with anti-oxidant and anti-inflammatory effects onto hollow cerium dioxide nanoparticles [35], which is expected to exert a synergistic effect in alleviating IBD symptoms, while exhibiting a more favorable safety profile compared with drug-loaded nanoparticle platforms. Therefore, the composite nanomaterials combining Res and CeO_2_ may be a potential solution. It both may improve the poor bioavailability of Res and complements the deficiencies of CeO_2_ in ROS scavenging. Additionally, Res demonstrates the capacity to regulate the gut microbiota and alleviate intestinal barrier function [23]. Thereby, this nanomaterial mitigates colitis via dual mechanism: combating oxidative stress and inflammation, and rectifying disruptions in the gut microbiota.

**Scheme 1:**
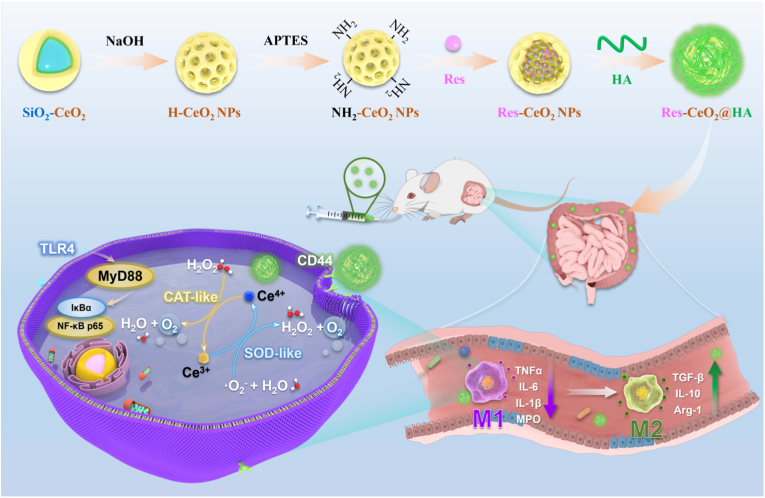
Schematic diagram in preparation of Res-CeO_2_@HA (A) and its therapy application in treating IBD by effectively clearing ROS, alleviating inflammation, and regulating gut microbiota.

In this study, the resveratrol-embedded hollow cerium oxide composite nanomaterials (Res-CeO_2_) was first designed. Res-CeO_2_ exhibit both SOD and CAT activities, serving as potent antioxidants to effectively scavenge ROS and as drug carriers to deliver Res. To address the challenges associated with oral administration and lack of targeting specificity, hyaluronic acid (HA) was modified on the surface of Res-CeO_2_ to form Res-CeO_2_@HA nanomedicine. HA is a polyanionic polysaccharide, which is commonly used as a carrier for targeted drug delivery [36]. Glycoprotein CD44 has been reported to be overexpressed on the surface of macrophages of patients with IBD, which contributed to the concentration of HA at the lesion regions in the colon [37]. Furthermore, the hyaluronidase degradation may not significantly influence the targeting capacity of HA based nanoparticle [38]. So, Res-CeO_2_@HA can specifically recognize and combine with overexpressed CD44 receptors from M1 macrophages [39], allowing it to rapidly enter the cells. It has excellent abilities in regulating inflammatory infiltration of macrophages, reducing the expression of pro-inflammatory cytokines by M1 macrophages, promoting polarization of M2 macrophages, and enhancing the secretion of anti-inflammatory cytokines. In addition, Res-CeO_2_@HA also could maintain a dynamic balance in the gut microbiota by regulating its structure and composition. Furthermore, the significant contribution of Res-CeO_2_@HA in alleviating IBD through the regulation of the TLR4/NF-κB signaling pathway was explored (Scheme 1). Compared with the single-functional material that alleviates IBD by removing ROS, Res-CeO_2_@HA demonstrates more pronounced efficacy. Li et al. [40] used the sodium ferulic-loaded hydrogel to adhere to the colonic mucosa and the results showed that the hydrogel persistently scavenged ROS in inflammatory areas and at the same time reduced the level of myeloperoxidase (MPO) by half. In our study, the MPO levels was decreased by two times and the improvement of colon length was more obvious in IBD mice. Similarly, the mRNA expression levels of anti-inflammatory cytokines were 2–4 times higher after treatment, while only about a 1-fold increase was observed in the Yan' s study [41]. Therefore, due to multifunctional and targeted characteristics, the nanomaterials have great potential in the treatment of IBD, and also provide scientific basis and evidence for the development of new nanomedicines for treating inflammatory diseases.

## 2 Experimental section

### 2.1 Regents

Ce(NO_3_)_3_·6H_2_O, Resveratrol (Res) and Hyaluronic acid (HA) were purchased from Macklin Biochemical Technology Co., Ltd (Shanghai, China). Dextran sulfate sodium salt (DSS) and 1,1′-dioctadecyl-3,3,3′,3′-tetramethylindotricarbocyanine iodide (DiR) were acquired from Yeasen Biotechnology Co., Ltd (Shanghai, China). Lipopolysaccharides (LPS), 2′, 7′-Dichlorofluorescin diacetate (DCFH-DA), Cell Counting Kit-8 (CCK-8) assay, enzyme linked immunosorbent assay (ELISA), myeloperoxidase (MPO), E.Z.N.A.® Soil DNA kit were bought from Aladdin Co., Ltd (Shanghai, China). Primary antibodies including MyD88 (bs-1047R), NF-κB p65 (bs-0465R), TLR4 (bs-20594R), CD86 (13395-1-AP-50), CD206 (18704-1-AP-50), ZO-1 (ab221547) and Occluding (ab216327) were obtained from Abcam Co., Ltd (MA, USA).

### 2.2 Synthesis of silica (SiO_2_)

The solid SiO_2_ was synthesized by Stöber's method [42]. First, 60 mL of ethanol, 8 mL of water and 4 mol/L of ammonia were mixed. Then, the mixture was stirred and heated to 60 °C. 10 mL of ethyl orthosilicate and 40 mL of ethanol were added to the above solution mixture and accompanied by heating and stirring for 4h. Finally, the SiO_2_ was dried after centrifugation and washing with water and ethanol.

### 2.3 Synthesis of hollow CeO_2_

According to the reported literature [43], 0.1 g of dried SiO_2_ and 1g of poly (vinylpyrrolidone) were dispersed in 40 mL of deionized water. Then, 10 mL of Ce(NO_3_). 6H_2_O (0.5 mM) and 10 mL of hexamethylene tetramine (0.5 mM) were added to the above solution and heated at 95 °C for 4 h. The precursor of SiO_2_@CeO_2_ was obtained after centrifugation and drying. Subsequently, the precursor was calcined at a high temperature of 600 °C for 4 h to obtain SiO_2_@CeO_2_. Then, 0.2 g of SiO_2_@CeO_2_ were added into 80 mL of NaOH solution (2 M) and stirred for 12 h to etch the SiO_2_ template. The hollow CeO_2_ was obtained after centrifugation and drying.

### 2.4 Synthesis of Res-CeO_2_ and Res-CeO_2_@HA

The Res-CeO_2_ and Res-CeO_2_@HA were prepared according to previous literature with slight modifications [44]. 5 mL of CeO_2_ solution (10 mg/mL) and 1 mL of aminopropyltriethoxysilane were uniformly dispersed into 44 mL of ethanol. The mixture was heated reflux for 12 h to form aminated CeO_2_. Then, Res (10 mg/mL) was added into the aminated CeO_2_ mixture with stirring for another 24 h to form Res- CeO_2_. Next, the HA solution activated by EDC and NHS was added to Res-CeO_2_ with stirring for 12 h. After centrifugation, Res-CeO_2_@HA was collected by washing and drying.

### 2.5 Characterization of Res-CeO_2_@HA

The morphology were characterized were obtained by HT7800 transmission electron microscope (TEM, Hitachi, Japan). The size and surface potential were quantified by nano ZS90 Zetasizer (Malvern, U.K.). The valence states were analyzed by Escalab 250Xi X-ray energy spectrometer (XPS, Thermo, USA). The X-ray diffraction pattern was analyzed by TD-3500 X-ray diffractometer (XRD, Tongda, China).The specific surface area was determined by ASAP 2460 automatic specific surface area analyzer (BET, Micromeritics, USA). Fourier transform infrared spectroscopy was conducted by FT-08 instrument (FTIR, Thermo, USA). UV–vis absorption spectra were collected by UV-2600 spectrophotometer (Shimadzu, Japan).

The encapsulation efficiency (EE) and drug loading (DL) of Res were calculated based on the following formulas. Calculation of EE and DL was based on Eqs. (1), (2), respectively:

(1) EE = Amount of Res loaded/Amount of Res added × 100 %

(2) DL = Amount of Res loaded/Total amount of Res-CeO_2_@HA harvested × 100 %

### 2.6 *In vitro* digestion assay

The digestive ability *in vitro* was evaluated by the simulated stomach–intestine digestion model. Briefly, 3 mL of Res-CeO_2_ and Res-CeO_2_@HA were added into dialysis bags (MW = 3.5 kDa), respectively. The dialysis bags were dialyzed in simulated gastric fluid (SGF, pH 2) for 2 h to simulate gastric digestion, and then transferred to simulated intestinal fluid (SIF, pH 7.2) for 6 h to simulate intestinal digestion. During the dialysis process, the release amount of Res was recorded every 0.5 h.

### 2.7 Cell culture and cytotoxicity assay

To evaluate the biocompatibility of Res-CeO_2_@HA, the cytotoxicity tests were conducted utilizing the CCK-8 assay with RAW264.7 cells. A series of concentrations of CeO_2_, Res-CeO_2_ and Res-CeO_2_@HA (25, 50, 75, 100, 200, 400 μg/mL) were firstly added into the cells and incubated for 24 h. After this, the viability of cells was evaluated using the CCK-8 assay kit. Additionally, cell apoptosis was analyzed using the Annexin V-FITC/PI Apoptosis Detection Kit (Vazyme, China).

### 2.8 Determination of NO production

The RAW264.7 cells were exposed to CeO_2_ and Res-CeO_2_@HA for 24 h, with the simultaneous addition of LPS solution (0.5 mg/mL). Following this, the supernatant from the cell culture medium was harvested for the determination of NO content using a specific NO detection kit.

### 2.9 Cellular ROS-scavenging assessment

To evaluated the protective of Res-CeO_2_@HA on cells from oxidative damage. 2′,7′-Dichlorofluorescin diacetate (DCFH-DA) was used to evaluate the intracellular total ROS levels. 0.5 mg/mL of LPS was added into RAW264.7 cells with different concentrations of CeO_2_, Res-CeO_2_ and Res-CeO_2_@HA for 24 h, and cells without LPS were used as the control group. The cells were then imaged using the FV-1000 confocal laser scanning microscope (Olympus, Japan).

### 2.10 Cell uptake

RAW264.7 cells were firstly induced with LPS solution (0.5 mg/mL) for 24 h. Then, the FITC-labeled Res-CeO_2_@HA were added into the mixture and incubated for 12 h. Subsequently, the cell nuclear was stained with 1X Hosechst 33342 for 10 min at 37 °C. After washing with PBS. The fluorescence staining of cells was observed. Meanwhile, the HA intervention group was conducted.

### 2.11 Anti-inflammatory assay *in vitro*

RAW264.7 cells were firstly induced with LPS solution (0.5 mg/mL) for 24 h, and followed by the addition of CeO_2_, Res-CeO_2_ and Res-CeO_2_@HA for another 24 h incubation. The supernatant was measured by ELISA kit. Relative mRNA expression of antioxidant genes was also measured by qRT-PCR assay. The primer sequences for the genes (TNF-α, IL-6, Arg-1, iNOS, TGF-β, IL-1β and IL-10) was presented in Table S1 in supporting information (SI). The parameters are set as follows for the qRT-PCR reaction: pre-denaturation at 95 °C for 180 s, denaturation at 95 °C for 30 s, annealing at 60 °C for 30 s, and extension at 72 °C for 30 s, for 40 cycles.

### 2.12 Macrophage polarization assay *in vitro*

RAW264.7 cells were blocked for 1 h with TBST buffer solution containing 1 % BSA and then incubated with CD86/CD206 antibodies overnight at 4 °C. After washing with PBS buffer solution, the cells were incubated with fluorescent secondary antibody for 1 h at 4 °C. Subsequently, the cells were collected and the expression of macrophage surface markers was measured by flow cytometry.

### 2.13 Dss-induced colitis model and treatment

Male Balb/c mice (6–8 weeks) were obtained from Henan Skobes Biotechnology Co., Ltd. The handling of mice shall comply with the “Henan Province Animal Experiment Management Regulations”. Furthermore, the experiments were performed in compliance with the guidelines formulated by Immunology Animal Laboratory of Henan Academy of Agricultural Sciences. The Balb/c mice were acclimatized for a week. DSS solution (3 %) was used to induce the ulcerative colitis mice model. Mice were randomly divided into six groups (n = 10 per group) including the control group fed with autoclaved purified water, DSS-induced colitis model group, colitis mice treated with 5-amino salicylic acid (5-ASA) (5-ASA group), CeO_2_ (CeO_2_ group), Res-CeO_2_ (Res-CeO_2_ group) and Res-CeO_2_@HA (Res-CeO_2_@HA group). 5-ASA (40 mg/kg), CeO_2_ (200 mg/kg), Res-CeO_2_ (200 mg/kg) and Res-CeO_2_@HA (200 mg/kg) was orally administered. After 7 days of on-demand feeding, the Disease Activity Index (DAI) for the colitis mice was evaluated based on three criteria (Table S2): stool consistency (rated 0–3), stool bleeding (rated 0–3), and weight loss (rated 0–4)

### 2.14 Anti-inflammatory effects *in vivo*

The MPO content of colon tissue was detected by MPO kit. The inflammation-related cytokines (IL-6, TNF-α and IL-10) in serum were measured by ELISA kit. The mRNA expression levels of key biomarkers in colon tissue was confirmed by qRT-PCR assay. The mRNA expression levels of M1 phenotype-related biomarkers (iNOS, IL-6, TNF-α and IL-1β) and M2 phenotype-related biomarkers (TGF-β and IL-10) were also measured.

### 2.15 Histopathological evaluation and immunofluorescence staining

The organs of mice (heart, liver, spleen, lung and kidney) were fixed with 4 % paraformaldehde solution and then embedded in paraffin. After slicing, the organ slices were stained with hematoxylin and eosin (H&E). The epithelium thickness and goblet cells of colon tissues were evaluated by periodic acid Schiff (PAS).The colon tissue embedded in paraffin were then received immunofluorescence staining. Sections were incubated with the antibodies of CD206, CD86, Occludin and ZO-1, respectively at 4 °C overnight, and then incubated with the corresponding fluorescent secondary antibody for 2 h at room temperature. The fluorescent images was captured by Ti-E inverted fluorescence microscope (Nikon, Japan).

### 2.16 Gut microbiota analysis

Mouse fecal DNA was extracted using the E.Z.N.A.® Soil DNA kit. The V3-V4 hypervariable regions of the 16S rDNA were amplified using the primers 338F-806R. Sequencing was performed on the Illumina MiSeq PE300 platform at Majorbio Bio-pharm Technology Co., Ltd (Shanghai, China). Furthermore, the intestinal microbiota data were visualized and analyzed using the online platform of I-Sanger Cloud Platform. Alpha diversity indices (Chao, Ace, Simpson, and Shannon), Venn diagrams, and species histograms at the phylum, family, and genus levels were utilized to evaluate the richness and diversity of the gut microbiota. Unweighted UniFrac Principal Coordinate Analysis (PCoA), Unweighted UniFrac Non-metric Multidimensional Scaling (NMDS), and Linear Discriminant Analysis (LDA) Effect Size (LEfSe) (LDA ≥4) were employed to assay the differences in structure and relative abundance of microbial communities. A phylogenetic investigation of communities by reconstruction of unobserved states (PICRUSt2) analysis was performed. Additionally, correlation Heatmap analysis was performed by calculating the correlation coefficients between clinical factors and selected species.

### 2.17 Statistical analysis

The data was reported as the mean ± standard deviation (S.D.). Statistical analysis was conducted using one-way ANOVA. Significant differences among the treated groups and control group were indicated as *P < 0.05, **P < 0.01, and ***P < 0.001. Significant differences among the treated groups and DSS-induced group were indicated as ^*#*^*P < 0.05,* ^*##*^*P < 0.01* and ^*###*^*P < 0.001*.

## 3 Results and discussion

### 3.1 Characterization of Res-CeO_2_@HA

**Fig. 1:**
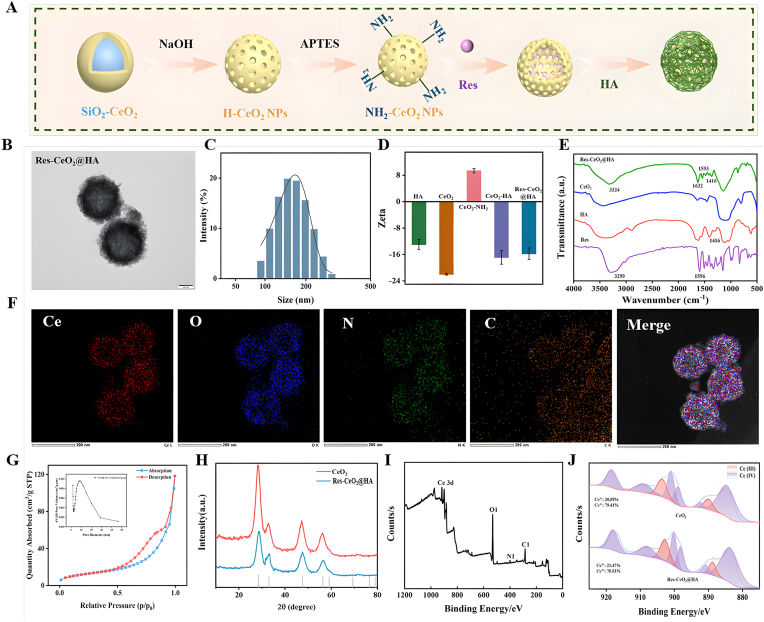
(A) Synthesis scheme of the Res-CeO_2_@HA. TEM images (B), Particle size distribution (C), Zeta potential (D), FT-IR spectra (E), Energy dispersive X-ray spectroscopy (EDS) elemental mapping images (F), N_2_ adsorption–desorption isotherm and BJH desorption dV/dD pore volume of CeO_2_ (G), X-ray diffraction patterns (XRD) (H), High-resolution XPS spectra (I), and XPS spectra of Res-CeO_2_@HA (J).

To construct Res-CeO_2_@HA (Fig. 1A), the hollow CeO_2_ was firstly prepared through a SiO_2_ template method. Then, Res was embedded in the hollow CeO_2_. Finally, HA was modified on the surface of Res-CeO_2_ to form Res-CeO_2_@HA. The TEM image indicated that Res-CeO_2_@HA presented a hollow spherical structure with coverings on its surface (Fig. 1B). Meanwhile, the morphology of SiO_2_ templates and hollow CeO_2_ were also confirmed by TEM in Figs. S1 and S2. The particle size of CeO_2_, Res-CeO_2_ and Res-CeO_2_@HA by DLS were also measured. It found that the average particle size of CeO_2_ was hardly changed after loading Res, the average particle size of CeO_2_ and Res-CeO_2_ was 132 nm ± 1.5 nm and 134 nm ± 1.4 nm, respectively (Fig. S3A and B), while the particle size of Res-CeO_2_@HA was increased significantly (150 nm ± 2.1 nm) after linking HA on the surface of CeO_2_ (Fig. 1C). As shown in Fig. 1D, HA with negatively charged was more easily modified on the surface of CeO_2_-NH_2_. Res was loaded onto CeO_2_-NH_2_ through electrostatic interaction, forming Res-CeO_2_ with a zeta potential of −2.01 mV ± 0.74 mV. The final Zeta potential of Res-CeO2@HA was −14.75 mV ± 1.16 mV. Previous study indicated that nanoparticles with a relatively high absolute value of negative charge tend to accumulate in colonic tissue [14]. Furthermore, the DL and EE were calculated as 6.17 % ± 0.32 % and 65.01 % ± 3.41 %, via the standard curve of Res obtained through UV–Vis spectrophotometry (Fig. S4A, B and C). The results were similar to Q@CeBG [45]. The characteristic peaks of HA, Res, CeO_2_-NH_2_ and Res-CeO_2_@HA were measured by FTIR. As shown in Fig. 1E, the peak of Res-CeO_2_@HA at 3324 cm^−1^ was the phenol absorption peak of Res, and the occurrence of tensile vibration near the characteristic peak 1596 cm^−1^ of the C=C group in Res indicated a successful loading of Res. The peak of Res-CeO_2_@HA at 1632 cm^−1^ was the characteristic absorption peak of the amide bond formed by the -COOH group and the amino group of HA [46], and the -COOH group of HA at 1416 cm^−1^ demonstrated the successful connection of HA.

Then, an energy spectrum analysis was continued on the basis of TEM to determine the structural features of Res-CeO_2_@HA. In Fig. 1F, the distribution of each element was matched the Res-CeO_2_@HA counterpart. The catalytic efficiency largely depends on the number of active sites present in nanoenzyme. In Fig. 1G, the BET surface area of CeO_2_ was measured to be 57.5592 m^2^/g based on N_2_ adsorption isotherm analysis. And the relationship between pore size and dV/dD pore volume verified that the pore channels in CeO_2_ was single-peaked mesoporous with the diameter around at 9.5 nm. It had been proved that the substantial specific surface area offered a sufficient foundation for the effective loading of CeO_2_ [47]. The crystal structures of CeO_2_ and Res-CeO_2_@HA were determined by XRD at a scattering angle of 0–80°. CeO_2_ exhibited the same diffraction curve as observed in previous report [47]. And the crystal structure of CeO_2_ was not changed after loading Res and attaching HA (Fig. 1H). As shown in Fig. 1I, Res-CeO_2_@HA had the peaks of Ce 3 d, O 1s, N 1s and C 1s. The high-resolution XPS spectra of Ce 3d in CeO_2_ and Res-CeO_2_@HA shown that the peaks located at 885.6 and 904.1 eV were belong to Ce^3+^, while the peaks located at 881.7, 898.1, 900.9, 906.4 and 916.4 eV were owing to Ce^4+^. And the content of Ce^3+^ and Ce^4+^ in CeO_2_ and Res-CeO_2_@HA was 20.59 % and 79.41 %, respectively (Fig. 1J). The results indicated that the mixed valence states of CeO_2_ demonstrated its excellent catalytic performance [48], and the formation of Res-CeO_2_@HA had not altered the mixed valence states of CeO_2_. Then, simulated digestion experiments were conducted to verify the digestive capabilities of Res-CeO_2_ and Res-CeO_2_@HA *in vitro*. In Fig. S5, after digesting in gastric fluid for 2 h, the release amounts of Res from Res-CeO_2_ and Res-CeO_2_@HA were 27.48 % and 17.52 %, respectively. The release rate of Res was gradually increased with the prolongation of digestion time. After 6 h of digestion, the release rates of Res from Res-CeO_2_ and Res-CeO_2_@HA were increased to 79.32 % and 57.83 %, respectively. The slower release rate of Res from Res-CeO_2_@HA indicated that the HA coating can protect Res from damage by gastrointestinal fluids. Zhang et al. [14] also found that HA can protect nanomaterials from damage by gastrointestinal fluids. Furthermore, TEM images of Res-CeO_2_@HA after digesting in gastric fluid for 2 h showed that its structure remained relatively intact (Fig. S6A). As shown in Fig. S6B, The changes in the particle size of Res-CeO_2_@HA during the simulated digestion process was further measured. The results indicated that the particle size of Res-CeO_2_@HA in gastric fluid showed no significant changes. However, a slight increasing trend in the particle size was observed in intestinal fluid as the incubation time continues to increase (Fig. S6B). This may be attributed to the partial degradation of hyaluronic acid by hyaluronidase in the intestinal fluid, leading to an increase in particle size. Additionally, the short-term stability studies revealed that the particle size of CeO_2,_ Res-CeO_2_ and Res-CeO_2_@HA did not change markedly within 25 d at room temperature (Fig. S7). This characteristic ensures its ability to target drug release and improve the absorption of drug after oral administration [49].

### 3.2 Biocompatibility and anti-inflammatory activity of Res-CeO_2_@HA

**Fig. 2:**
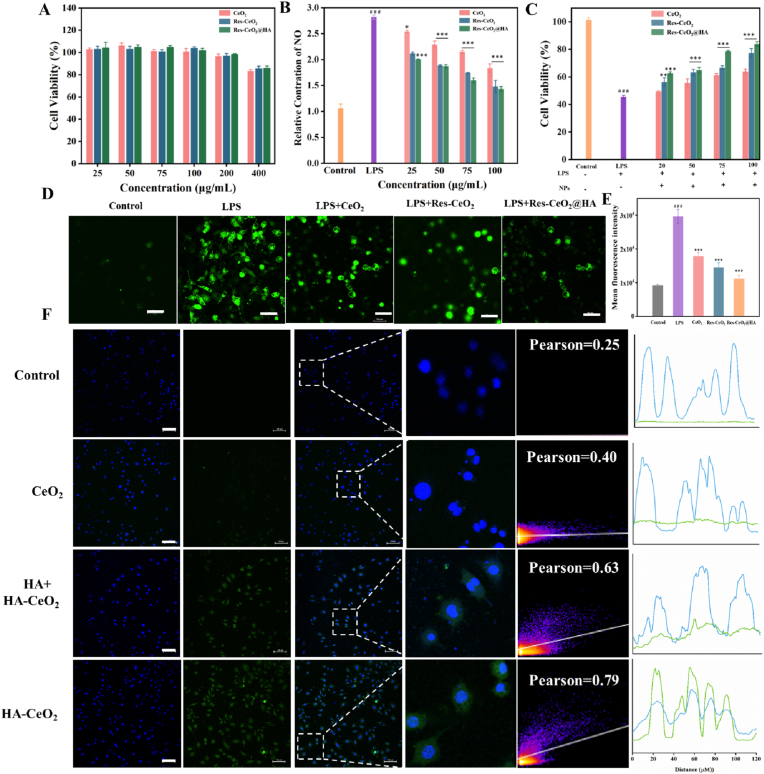
Biosafety and ROS-scavenging activities and Cell uptake *in vitro*. (A) Cell viability of RAW264.7 cells after treatment with different concentrations of Res-CeO_2_@HA. (B) Cell viability and (C) NO levels of RAW264.7 cells after treatment with LPS-induced and different concentrations of CeO_2_, Res-CeO_2_ and Res-CeO_2_@HA. (D) CLSM images and (E) Fluorescent signal histogram indicating the ROS production in RAW264.7 cells after treatment with CeO_2_, Res-CeO_2_ and Res-CeO_2_@HA, scale bar = 50 μm. (F) CLSM images of cell uptake of Res-CeO_2_@HA, scale bar = 100 μm. Data are mean ± SD (n = 3).

The biocompatibility of Res-CeO_2_@HA was evaluated by CCK-8 assay. As shown in Fig. 2A, the RAW264.7 cells exhibited good viability even exposed to CeO_2_, Res-CeO_2_, and Res-CeO_2_@HA at a concentration of 400 μg/mL for 24 h, indicating that the excellent biocompatible and non-toxic characteristics of Res-CeO_2_@HA to RAW264.7 cells. Lipopolysaccharide (LPS), a classic endotoxin derived from the outer membrane of Gram-negative bacteria, potently induces ROS production and inflammatory responses in the host [50]. Therefore, the anti-inflammatory activity can be validated by assessing the improvement in the cell viability of LPS-induced cells *in vitro*. In Fig. 2C, after stimulation of LPS, the cells viability significantly decreased to 47 %. However, the cells viability was increased significantly after treatment with CeO_2_, Res-CeO_2_ or Res-CeO_2_@HA. When the concentration of nanomedicines were 100 μg/mL, the cell viability in the Res-CeO_2_@HA group was reached 85 %, which was higher than that in Res-CeO_2_ group (77 %) and CeO_2_ group (60 %). To further validate the mitigating effect of Res-CeO_2_@HA on cellular inflammation, the Annexin V-FITC/PI staining was performed. Compared to the control group, the number of viable cells was significantly decreased after LPS induction. However, following the treatment of CeO_2_, Res-CeO_2_ and Res-CeO_2_@HA, the cells viability was improved markedly, indicating that the nanomedicines had protective effects against the LPS-induced damage. The Res-CeO_2_@HA showed best therapeutic effect than CeO_2_ and Res-CeO_2_ (Fig. S8), which may due to the anti-inflammatory property of HA and Res in the Res-CeO_2_@HA [51]. Furthermore, the inducible nitric oxide synthase (iNOS) that formed in cells stimulated by LPS subsequently released NO, serving as a marker for evaluating the inflammatory status of the cells [52]. In Fig. 2B, the NO content of cells induced by LPS was significantly increased compared with control group (p < 0.001), with a relative content reaching approximately 2.7 ± 0.5. However, after treatment with CeO_2_, Res-CeO_2_ and Res-CeO_2_@HA, the relative NO content was significantly reduced compared with LPS-induced group (p < 0.001), showing a concentration-dependent effect. In Res-CeO_2_@HA (100 μg/mL) treatment group, the intracellular NO content was decreased to approximately 1.5 ± 0.8, comparable to the NO content in the control group. It was confirmed by Kobyliak et al. in their exploration of the anti-inflammatory of CeO_2_ in alleviating nonalcoholic fatty liver disease [53].

Next, the early apoptosis of cells was assessed by measuring the mitochondrial membrane potential (MMP) using the JC-1 probe. As shown in Fig. S9, the control group showed strong red fluorescence. In contrast, the LPS-treated group exhibited weak red fluorescence and strong green fluorescence, indicating the decreased MMP and early apoptosis in RAW264.7 cells [54]. After incubation with CeO_2_, Res-CeO_2_ and Res-CeO_2_@HA for 24 h, the red fluorescence of RAW264.7 cells was significantly enhanced, and the green fluorescence was weakened, indicating that the nanoparticles could reduce the decrease of MMP and inhibit LPS-induced inflammatory damage [55]. Notably, the red fluorescence of Res-CeO_2_@HA group was significantly stronger than that of CeO_2_ or Res-CeO_2_ group.

### 3.3 ROS scavenging capacity of Res-CeO_2_@HA

The ROS scavenging abilities of Res-CeO_2_@HA towards DPPH, •OH, •O_2_- and H_2_O_2_ were investigated, with CeO_2_ and Res-CeO_2_ serving as controls. As shown in Fig. S10A and B, as the concentration increased, the DPPH and •OH scavenging rates of CeO_2_, Res-CeO_2_ and Res-CeO_2_@HA gradually increased. At a concentration of 100 μg/mL, the clearance rates of •OH and DPPH by Res-CeO_2_@HA was reached 47 % and 54 % respectively, and its effect was superior to that of free CeO_2_ at the same concentration. However, the effect was less pronounced than that of Res-CeO_2_, which could be attributed to the higher Res release rate of Res-CeO_2_ [56]. The same results were obtained when measuring the scavenging rate of •OH radicals using paramagnetic assay (Fig. S11). SOD is an enzyme that catalyzes the dismutation of •O_2_^−^ into H_2_O_2_ and O_2_, providing protection against cellular oxidative stress [57]. The SOD-like catalytic activity of Res-CeO_2_@HA was conducted using the WST-8 method. In Fig. S12A, Res-CeO_2_@HA could effectively scavenge •O_2_^−^ in a dose-dependent manner, and the scavenging rate has reached 45 % at a concentration of 100 μg/mL. The H_2_O_2_ generated from the •O_2_^−^ dismutation reaction was further decomposed into H_2_O and O_2_ by CAT. The CAT-like catalytic activity was also evaluated by measuring the scavenging rate of H_2_O_2_. Res-CeO_2_@HA had the higher scavenging efficiency compared to free CeO_2_. Similarly, the scavenging efficiency of Res-CeO2@HA is higher than that of CeO_2_, but lower than that of Res-CeO_2_ (Fig. S12B). As shown in Fig. S13, the scavenging capacity of Res was increased gradually with the rise in concentration. At a concentration of 6 μg/mL, the DPPH scavenging rate reached 25 %. However, its scavenging effects on H_2_O_2_ and •O_2_^−^ were weaker than that on DPPH. Furthermore, the ROS clearance ability of CeO_2_, Res-CeO_2_ and Res-CeO_2_@HA were estimated by CLSM in RAW264.7 cells. As shown in Fig. 2D and E and Fig. S14, after LPS-induced treatment, the intracellular fluorescence intensity that captured by DCFH-DA kit was significantly improved compared with the control group (p < 0.001), indicating an exacerbation of oxidative stress. However, after intervention with the CeO_2_, Res-CeO_2_ and Res-CeO_2_@HA at different concentrations, the green fluorescence was weakened compared to LPS-induced group (p < 0.001). Among them, the Res-CeO_2_@HA group exhibited the best effect, demonstrating its powerful ROS scavenging capability.

### 3.4 Cell uptake ability of Res-CeO_2_@HA

Efficient cellular-targeted uptake of Res-CeO_2_@HA is another crucial factor in treating inflammation. Considering that HA is a specific ligand for the LPS-induced high expression of CD44 receptor on the surface of RAW264.7 cells, FITC-HA-CeO_2_ was prepared with FITC instead of Res. In Fig. 2F, a weak green fluorescence of FITC was observed in cells treated with CeO_2_. While the green fluorescence was obviously observed in cells after treatment with FITC-HA-CeO_2_. The scatter plot of cells treated by FITC-HA-CeO_2_ deviated from the diagonal and indicated a scattered distribution. Compared with the FITC-CeO_2_ group (0.40 ± 0.01),the total value of the FITC-HA-CeO_2_ group (0.79 ± 0.02) was remarkably higher. In addition, the competition treatment with free HA was carried out to verify the cellular uptake caused by HA. The cells were treated with HA for 2 h and then added with FITC-HA-CeO_2_. As a result, because of the HA competition, the cellular uptake of FITC-HA-CeO_2_ was significantly reduced, the total value decreased to 0.63 ± 0.04. Furthermore, the uptake efficiency of CeO_2_, HA + HA-CeO_2_, and HA-CeO_2_ was analyzed by flow cytometry, which confirmed the preferential macrophage-targeting capacity of HA-CeO_2_ (Fig. S15). Li et al. [26] also found that HA can enhance cellular uptake. Gao et al. utilized HA to modify CeO_2_, thereby enabling it with targeting properties [58]. These results suggested that Res-CeO_2_@HA had good targeting ability towards macrophages.

### 3.5 Anti-inflammatory effects of Res-CeO_2_@HA *in vitro*

**Fig. 3:**
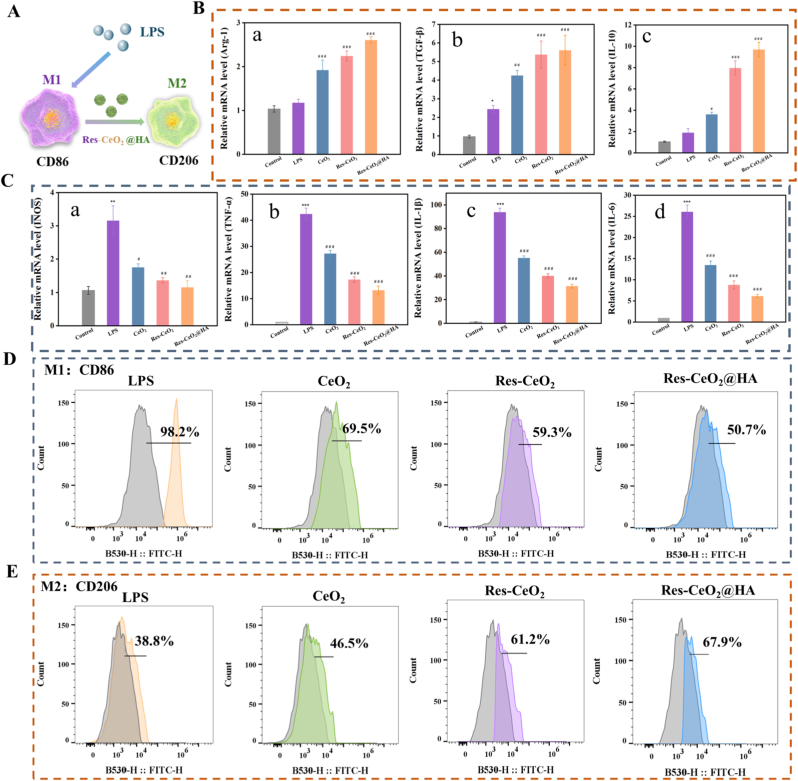
Macrophage polarization of Res-CeO_2_@HA *in vitro*. (A) Image illustration of Res-CeO_2_@HA regulating macrophage polarization. (B) In vitro mRNA expression of anti-inflammatory biomarkers (Arg-1, TGF-β and IL-10) and (C) pro-inflammatory cytokines (TNF-α, IL-6, iNOS and IL-1β) with LPS-induced, CeO2, Res-CeO2 and Res-CeO_2_@HA treatment. (D) M1 macrophage CD86 and (E) M2 macrophage CD206 with different experimental. Data are mean ± SD (n = 3).

The anti-inflammatory capacity of Res-CeO_2_@HA can be assessed through macrophage polarization. The RAW264.7 cells was first transformed into M1 macrophages with the stimulation of LPS (Fig. 3A). M1 macrophages, also referred to as activated macrophages, primarily contribute to the defense against exogenous pathogens through the secretion of various pro-inflammatory cytokines and generation of ROS [59]. Inflammatory macrophages are activated by binding with TLR4 on the macrophages after LPS induction, which activate nicotinamide adenine dinucleotide phosphate oxidase (NOX2) to trigger respiratory burst and form superoxide anion radicals [60]. The accumulation of ROS enhances cellular oxidative stress response, resulting in increased inflammation. Overproduction of M1 macrophages can result in a series of inflammatory conditions [61]. In Fig. 3C, the mRNA expression of pro-inflammatory cytokines associated with M1 macrophages including iNOS, TNF-α, IL-1β and IL-6 were investigated by RT-qPCR. The expression of these related inflammatory cytokines was significantly increased after LPS induction, confirming the successful establishment of an inflammation model *in vitro* [62]. Compared with LPS-induced group, the CeO_2_, Res-CeO_2_ and Res-CeO_2_@HA treatment diminished the secretion of inflammatory cytokines notably. Res-CeO_2_ and Res-CeO_2_@HA treatment group showed more distinct inhibitory effects than that of CeO_2_ treatment group, demonstrating the synergistic action of Res and CeO_2_. Furthermore, the effect of Res-CeO_2_@HA was found to be more significant than that of Res-CeO_2_. This was due to the targeting effect of HA, which improved the cellular uptake capacity. Additionally, the mRNA expression of anti-inflammatory cytokines including TGF-β, Arg-1 and IL-10 which associated with M2 macrophages were also detected. The LPS-stimulated group failed to increase their expression compared to control group. However, the anti-inflammatory cytokines were all significantly increased after intervention with CeO_2_, Res-CeO_2_ and Res-CeO_2_@HA (Fig. 3B).

To assess the capability of Res-CeO_2_@HA in promoting macrophage polarization, CD86 and CD206 (the surface marker of M1 and M2 macrophages, respectively) were identified by flow cytometry. The results demonstrated that the expression of CD86 was significantly increased to 98 % after LPS induction. However, following intervention with CeO_2_, Res-CeO_2_ and Res-CeO_2_@HA, the expression of CD86 was decreased markedly. And Res-CeO_2_@HA exhibited better effects compared to that of CeO_2_ and Res-CeO_2_ (Fig. 3D and Fig. S16A). Similarly, compared with the LPS-stimulated group (38.8 %), the expression of CD206 was significantly increased after intervention with CeO_2_, Res-CeO_2_ and Res-CeO_2_@HA. And the Res-CeO_2_@HA group was increased to 67.9 % (Fig. 3E and Fig. S16B). Similarly, Chen et al. reported that Ce-Cur significantly inhibits the polarization of M1 macrophages compared with the Ce group [63]. These results indicated that Res-CeO_2_@HA could significantly enhanced the polarization of cells towards the M2 phenotype. Moreover, the contents of TNF-α, IL-1β, and IL-6 in LPS group were significantly higher than that of CeO_2_, Res-CeO_2_ and Res-CeO_2_@HA groups (Fig. S17). In contrast, after incubation with CeO_2_, Res-CeO_2_ and Res-CeO_2_@HA, the secretion of these pro-inflammatory cytokines was decreased, indicating that these nanoparticles had an anti-inflammatory effect. In addition, the content of IL-10 was increased in cells treated with CeO_2_, Res-CeO_2_ and Res-CeO_2_@HA (Fig. S18). Therefore, the Res-CeO_2_@HA showed good anti-inflammatory properties.

### 3.6 Therapeutic effect of Res-CeO_2_@HA on DSS-induced IBD mice *in vivo*

**Fig. 4:**
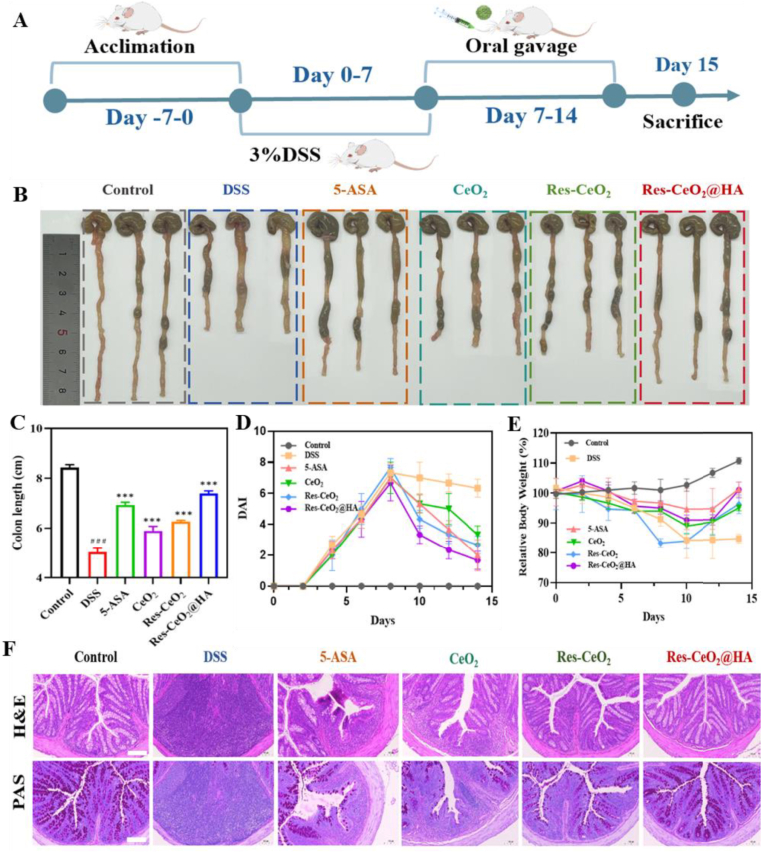
Therapeutic effects of Res-CeO_2_@HA on colitis. (A) Schematic diagram of colitis mice induced with 3 % DSS. (B) Optical photographs of colons (n = 3). (C) Colon length, (D) Body weight, (E) DAI scores of healthy control, DSS control, 5-ASA, CeO_2_, Res-CeO_2_ and Res-CeO_2_@HA treatment groups (n = 6). (F) H&E and PAS staining with different treatment, scale bare = 100 μm.

Due to the biocompatibility, antioxidant and anti-inflammatory of Res-CeO_2_@HA, the therapeutic effect of Res-CeO_2_@HA on DSS-induced IBD mice was further conducted (Fig. 4A). As an important indicator in the development of colitis, the change of colon length was investigated firstly [64]. In Fig. 4B and C, the colon in the control group was intact with a smooth surface and measured approximately 8.5 cm in length. While, the DSS group had a significantly shortened colon length of about 4.5 cm and appeared noticeably red. After treatment with CeO_2_, Res-CeO_2_ and Res-CeO_2_@HA, a remarkably increase in colon length was observed. Specifically, the colon length of the Res-CeO_2_@HA group was approximately 7.5 cm. And its therapeutic effect was confirmed by comparing with 5-ASA, a clinically used therapeutic agent for colitis [9]. As shown in Fig. 4D, the body weight of colitis mice was decreased significantly, while there was a noticeable improvement in body weight after intervention with Res-CeO_2_@HA for 12 days. The Disease Activity Index (DAI) results also clearly showed that intervention with 3 % DSS solution led to significant fecal bleeding, weight loss and diarrhea in mice. Res-CeO_2_@HA treatment showed a decrease in DAI, gradually alleviating the symptoms (Fig. 4E). The colonic crypt is a small concave structure located on the surface of the colonic mucosa, containing a large number of stem cells and secretory cells that regulate the balance of local immune responses in the intestine [65]. Studies have shown that DSS-treated mice exhibit significant ulceration in the colon, with extensive tissue destruction, disappearance of colonic crypt structure, a large number of goblet cell losses, and severe lamina propria damage [66]. Therefore, H&E and PAS staining were further carried out to explore the inflammatory damage in colonic tissues. In Fig. 4F, DSS-induced mice exhibited significant tissue edema with severe destruction of mucosal epithelial cells, disappearance of goblet cells, and massive infiltration of inflammatory cells in the colon. Res-CeO_2_@HA treatment restored colon length and alleviated microstructural damage, demonstrating therapeutic effects on IBD. In addition, the spleen swelling has slightly improved (Fig. S19). And there were no notable pathological abnormalities or impairments observed in the primary organs that exposed to Res-CeO_2_@HA (Fig. S20). The potential systemic effects of Res-CeO_2_@HA were further assessed by measuring serum markers for liver function (AST and ALT), kidney function (BUN) and blood index parameters (red blood cells (RBC), hemoglobin (HGB), mean platelets (PLT) and white blood cells (WBC)) (Fig. S21A–G). The results showed the good biocompatibility and low toxicity of Res-CeO_2_@HA [67].

Besides, as shown in Fig. S22A and 22B, the intracellular SOD and CAT activities were increased significantly compared with the DSS group. In Fig. S22C, IBD mice produced the excessive accumulation of MDA. Compared with the DSS-induced group, the production of MDA was decreased after pretreatment with Res-CeO_2_@HA. MPO activity is associated with neutrophil infiltration [68], therefore, MPO activity was also detected to verify the therapeutic efficacy of Res-CeO_2_@HA. In Fig. S23A, the MPO activity in DSS-induced group was elevated compared with control group (p < 0.001). While the MPO activity after Res-CeO_2_@HA treatment was reduced (p < 0.001), approaching the level similar to control group. The findings proved that Res-CeO_2_@HA could decrease MPO activity and reduce inflammatory response, thereby alleviating colonic inflammation. Similarly, the level of ROS in colonic tissue showed the same trend (Fig. S23B). The levels of pro-inflammatory cytokines (IL-1β, IL-6, and TNF-α) were decreased, whereas the level of anti-inflammatory cytokine (IL-10) was increased in Res-CeO_2_@HA treatment group (Figs. S24–25). All the findings suggested that Res-CeO_2_@HA were capable of effectively mitigating DSS-induced colitis by decreasing the influx of inflammatory cells and secretion of pro-inflammatory cytokines.

### 3.7 Effect of Res-CeO_2_@HA on the colonic barrier in IBD mice

**Fig. 5:**
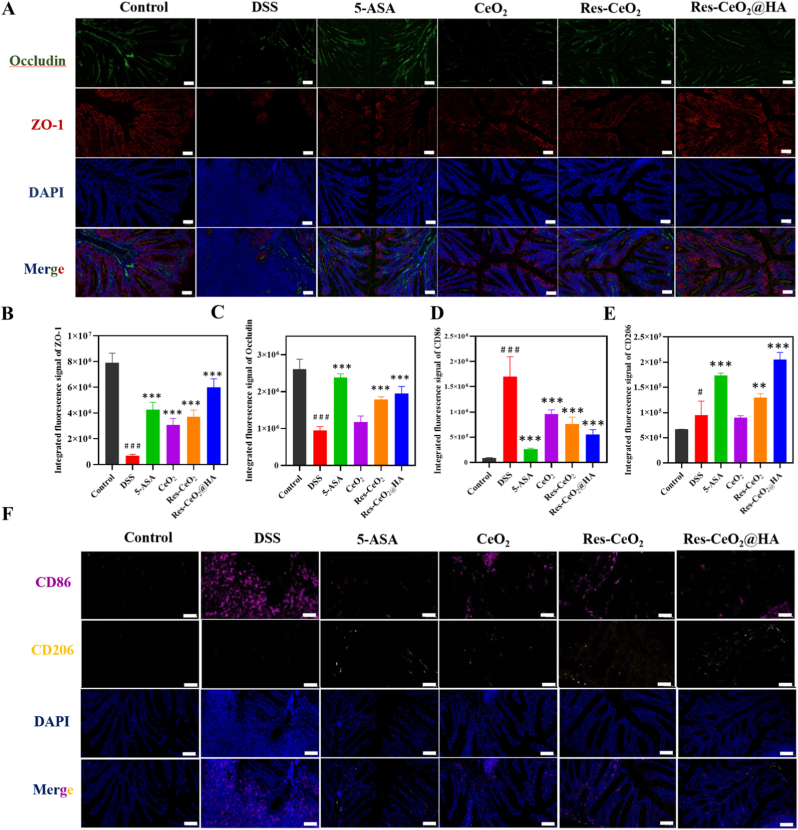
Intestinal barrier of Res-CeO_2_@HA on DSS-induced colitis *in vivo*. (A–C) Immunofluorescence and quantitative analysis of ZO-1 and Occludin in different treatment groups. (D–F) Quantitative analysis of immunofluorescence CD86 and CD206 and immunofluorescence of CD86 and CD206 (F) in different treatment groups. Data are mean ± SD (n = 3).

Occludin and ZO-1 are tight junction proteins within intestinal epithelial cells that play pivotal roles in maintaining the structural integrity and functional normality between cells. They serve as connectors linking membrane proteins to the cytoskeletal framework [69]. Therefore, the content of Occludin and ZO-1 in tissues can reflect the integrity of intestinal physical barrier function [70]. Hence, the immunofluorescence was conducted on paraffin sections of colon tissues to measure the content of Occludin and ZO-1 in different treatment groups. In Fig. 5A–C, the edges of the colonic epithelium of healthy mice (control group) were smooth and flat, with tight junction proteins primarily expressed in colonic epithelial cells, particularly at the apical junctions of colonic mucosal epithelial cells, where the expression levels were relatively high. The group of mice induced by DSS exhibited colonic epithelial mucosal erosion with uneven distribution, leading to a decrease in the expression of Occludin and ZO-1. After treatment with Res-CeO_2_@HA, the edges of the colonic epithelium of mice were smooth and flat, with the two tight junction proteins primarily expressed in colonic epithelial cells. Pan et al. had found that Res alleviated colitis by mitigating intestinal barrier dysfunction [71]. These results indicated that intervention with Res-CeO_2_@HA could enhance the expression of tight junction proteins and integrity of intestinal barrier function.

### 3.8 Effect of Res-CeO_2_@HA on macrophage polarization

Macrophages serve as gatekeepers of intestinal immune homeostasis. The sign of IBD is the loss of tolerance to symbiotic bacteria and food antigens caused by a decrease in intestinal macrophages [72]. Therefore, the infiltration of macrophages in colon was quantified by immunofluorescence staining. M1 and M2 macrophages were firstly labeled with CD86 and CD206 marker, respectively. In Fig. 5D–F, there was a prominently increase in macrophage infiltration in DSS-induced mice group. After Res-CeO_2_@HA treatment, the number of M1 macrophages was declined while the number of M2 macrophages was improved significantly. Zhang et al. discovered that polydopamine-modified CeO_2_ alleviated colitis inflammation by modulating M2 polarization of macrophages [73]. Ding et al. also drew similar conclusions [74]. These results indicate that Res-CeO_2_@HA can modulate the M1/M2 ratio of macrophages, thereby enhancing M2 polarization. Next, the effect of Res-CeO_2_@HA on inflammation-related factors in colonic tissue was evaluated through RT-qPCR. As shown in Fig. S26A–D, after intervention with Res-CeO_2_@HA, the mRNA level of pro-inflammatory cytokines including IL-1β, TNF-α, iNOS and IL-6 linked to M1 macrophages were markedly reduced. In contrast, the mRNA level of the anti-inflammatory cytokine including IL-10 and TGF-β associated with M2 macrophages were notably enhanced compared with DSS-induced group (p < 0.001) (Fig. S27). The results proved that Res-CeO_2_@HA had the potential to shift macrophage polarization towards the M2 phenotype in inflammatory tissues, thereby protecting the intestinal mucosa.

### 3.9 Effect of Res-CeO_2_@HA on TLR4/NF-κB signaling pathway in the colon of IBD mice

**Fig. 6:**
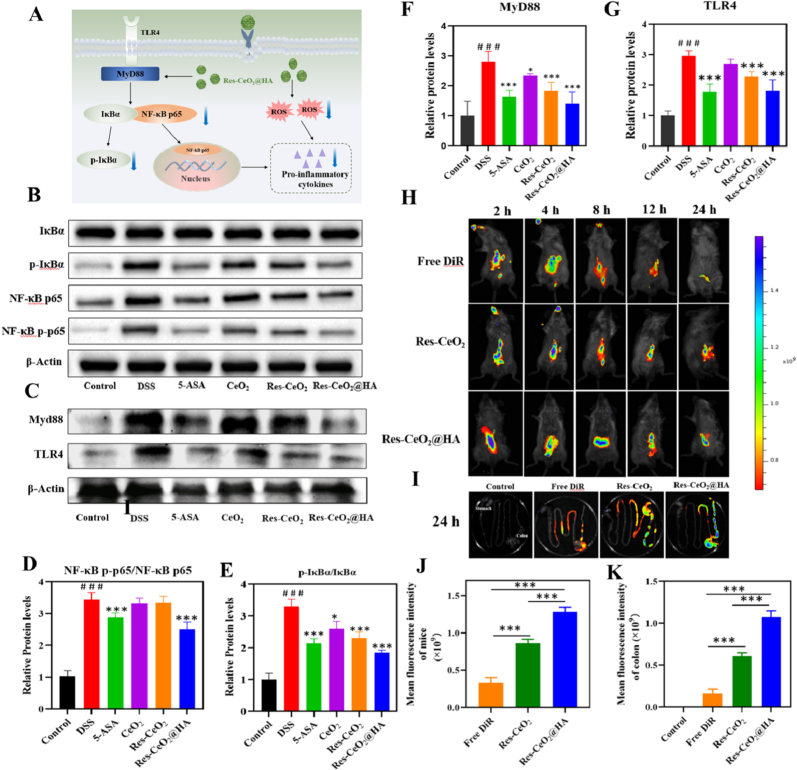
(A) Schematic diagram of Res-CeO_2_@HA regulating the signaling pathway. WB images (B–C) and Protein expression levels (D–G) of IκBα, p-IκBα, NF-κB p65, NF-κB p-p65, MyD88 and TLR4 in different treatment groups. (H) Fluorescence images of IBD mice after treatment with free DiR, DiR labeled on Res-CeO_2_ and Res-CeO_2_@HA at different time points. (I) Fluorescence images of IBD mice colon after treatment with free DiR, DiR labeled on Res-CeO_2_ and Res-CeO_2_@HA at 24 h. (J–K) Quantitative analysis of mice and colon. Data are mean ± SD (n = 3).

The TLR4/NF-κB signaling pathway mediates inflammatory responses in IBD. TLR4 acts as the recognition receptor that initiates the downstream inflammatory signaling cascade of NF-κB through the recruitment of MyD88. Under normal physiological conditions, NF-κB binds to the nuclear factor-kappa B inhibitor (IκB) and remains in the cytoplasm in an inactive form. Upon stimulation, IκB becomes phosphorylated, leading to its dissociation from NF-κB, which subsequently translocates from the cytoplasm to the nucleus, assisting in upregulating the expression of pro-inflammatory cytokines and exacerbating the severity of IBD (Fig. 6A) [75,76]. Therefore, the typical proteins were analyzed by Western blotting (WB) to evaluate the role of Res-CeO_2_@HA in the TLR4/NF-κB signaling pathway. In Fig. 6B–G, the total protein expression levels of TLR4 and MyD88 and the ratio of NF-κB p-p65/NF-κB p65 and p-IκBα/IκBα in DSS-induced group were obviously improved compared with control group (p < 0.001). After treatment with Res-CeO_2_@HA, the protein expression level of TLR4 and MyD88, and the phosphorylation of NF-κB p65 and IκBα were all remarkably reduced. The results indicated that Res-CeO_2_@HA exerted an anti-inflammatory activity by modulating the TLR4/MyD88/NF-κB signaling pathway [77]. To visually observe the biodistribution and targeting ability of Res-CeO_2_@HA, DiR was used to fluorescently label the Res-CeO_2_@HA. After treated with 24 h, the fluorescence in mice and colonic tissues was observed. In Fig. 6H and J, no significant fluorescence was observed in mice of free DiR group, while obvious fluorescence was detected in both Res-CeO_2_ group and Res-CeO_2_@HA. Furthermore, Res-CeO_2_@HA group exhibited higher fluorescence intensity, which was predominantly distributed in the colon, as confirmed by fluorescence imaging of the colon tissue (Fig. 6I and K). In addition, to further investigate whether Res-CeO_2_@HA can be preferentially taken up by macrophages in the colon tissues, sections are stained with FITC-conjugated F4/80 Abs. The red fluorescence of the Res-CeO_2_@HA group was clearly distributed in the colonic tissue, whereas it was markedly diminished in the Res-CeO_2_ group. It was confirmed that oral administration of Res-CeO_2_@HA enables its delivery to the colon and its absorption by macrophage (Fig. S28A–B). These findings can be ascribed to the upregulation of CD44 receptors at the site of inflammation, coupled with the increased accumulation of Res-CeO_2_ at this location due to the incorporation of HA, which facilitates interaction with CD44 expressed at the inflammatory site [78].

### 3.10 Regulatory effect of Res-CeO_2_@HA on the intestinal flora

**Fig. 7:**
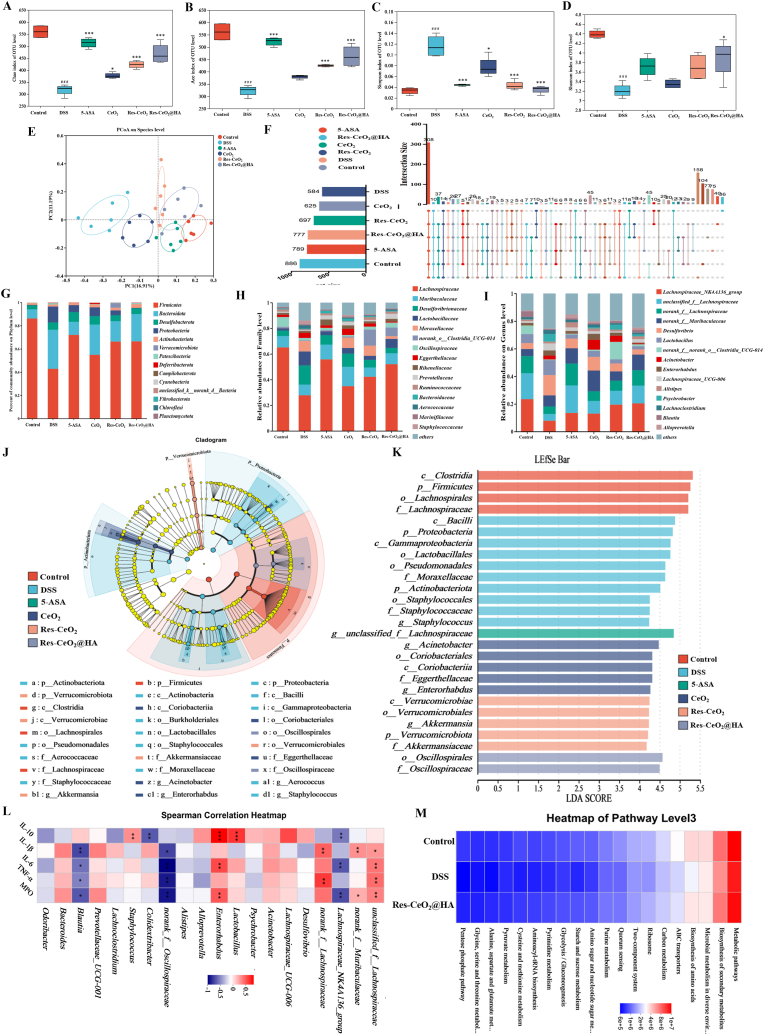
Diversity and composition analysis of gut microbiota with different treatment groups. (A–D) α-diversity analysis of Chao index, ACE index, Simpson index, and Shannon index. (E) Principal co-ordinates analysis on species level. (F) UpSet Venn diagram of gut microbiota species at OTUs level. The relative abundance histogram of species at (G) Phylum level, (H) Family level, and (I) Genus level. LefSe analysis (J) and (K) Linear discriminant analysis (LDA score≥4). (L) Correlation between IBD-related indices and gut microbiota composition at genu level. (M) Functional prediction of KEGG pathways (KEGG level 3; top 20) by PICRUSt2 analysis. Data are mean ± SD (n = 6).

Increasing studies indicated that dysbiosis of the gut microbiota is one of the primary culprits behind the progression of IBD [79]. Hence, ensuring the stability of gut microbiota is one of the crucial measures for treating IBD. The regulatory ability of Res-CeO_2_@HA on gut microbiota was evaluated by 16S rRNA sequencing analysis. Colitis induced alterations in the diversity and richness of microbial communities in colon tissue, as reflected in α-diversity metrics including Chao1, Ace, Shannon and Simpson. In Fig. 7A and B, both Chao1 and Ace indices in Res-CeO_2_@HA treatment group were significantly increased compared that with DSS-induced group, suggesting that Res-CeO_2_@HA can significantly improve the richness of gut microbiota in mice. Similarly, Res-CeO_2_@HA treatment significantly remarkably increased the Shannon index and decreased the Simpson index, demonstrating its ability to regulate gut microbial community diversity (Fig. 7C and D). Subsequently, the impact of Res-CeO_2_@HA on β-diversity of gut microbiota was analyzed by Principal Co-ordinates Analysis (PCoA) and Non-Metric Multidimensional Scaling (NMDS). In Fig. 7E and Fig. S29, The PCoA and NMDS analysis results of Res-CeO_2_@HA revealed that its microbial community species overlap with those of the control group, demonstrating its potential ability to improve microbial diversity. In addition, the changes of composition of gut microbiota by Res-CeO_2_@HA were also investigated. The number of species such as operational taxonomic units (OTUs) shared among the groups were observed by UpSet Venn diagrams. As shown in Fig. 7F, the Res-CeO_2_@HA treatment group had a total of 777 species, which was closed to 5-ASA treatment group (789 species). It had a significant improvement compared to DSS-induced group, once again proving the ability of Res-CeO_2_@HA to improve gut microbiota diversity. Furtherly, at phylum level, after intervention with Res-CeO_2_@HA, the relative abundance of *Bacteroidetes* and *Proteobacteria* was reduced while that of *Firmicutes* was raised compared to the DSS-induced group (Fig. 7G, Fig. S30 and Fig. S31). As two significant bacterial phyla, it had been proved that the increase in relative abundance of *Firmicutes* while the decrease in that of *Bacteroidetes* can effectively alleviate colitis [80]. Li et al. also found that Res could alleviate colitis symptoms in mice by increasing the ratio of *Firmicutes* to *Bacteroidetes* [81]. At the family level, Res-CeO_2_@HA treatment triggered a marked enhancement in the relative abundances *Lachnospiraceae* while a decrease in *Desulfovibrionaceae* and *Moraxellaceae* (Fig. 7H, Fig. S32 and Fig. S33). *Lachnospiraceae* can promote the repair and integrity of intestinal mucosa by producing substances such as short-chain fatty acids, thereby preventing harmful substances from entering the body to induce inflammation [80]. It also could inhibit inflammatory responses by regulating the immune system [81]. Additionally, both *Desulfovibrionaceae* and its flagellar protein can promote the initiation and progression of inflammation [82]. For instance, *Desulfovibrionaceae* can exacerbate intestinal inflammation in mouse models of ulcerative colitis [83]. *Desulfovibrionaceae* flagellar protein induces macrophage apoptosis by activating the NAIP/NLRC4 inflammasome, thereby promoting intestinal inflammation *in vitro* [84]. At the genus level, the relative abundance of *Lachnospiraceae_NK4A136_*group was increased while that of *norank_f__Muribaculaceae* and *Desulfovibri* was decreased in Res-CeO_2_@HA treatment group (Fig. 7I, Fig. S34 and Fig. S35). These findings indicate that the treatment with Res-CeO_2_@HA altered the composition of gut microbiota with increased abundance of beneficial bacteria, and shifted the gut microbiota composition towards that of the control group.

Furthermore, the impact of Res-CeO_2_@HA on the inter-group differences in gut microbiota species in mice was analyzed by Linear discriminant analysis Effect Size (LEfSe). As shown in Fig. 7J and K, the LEfSe bar plot displayed species with Linear Discriminant Analysis (LDA) scores exceeding the set threshold, indicating statistically significant differences among groups. An enhance in the relative abundance of *Akkermansiaceae* and *Oscillospirales* was observed in colon cells after Res-CeO_2_@HA treatment. Simultaneously, the relative abundance of *Proteobacteria* and *Bacilli* were notably decreased. Zhang et al. [85] demonstrates that probiotic *Akkermansiaceae* can ameliorate DSS-induced colonic inflammation in mice by improving intestinal permeability and enhancing the intestinal barrier function. Liu et al. [86] findings indicate that certain strains of *Akkermansiaceae* may mitigate colitis through the promotion of IL-22 secretion. Hence, Res-CeO_2_@HA treatment suppressed the relative abundance of pathogenic bacteria and improved the abundance and diversity of beneficial bacteria, thereby improved the dysregulation of the intestinal microbiota caused by IBD [63,64]. These functions may be owing to the antioxidant and anti-inflammatory properties of Res-CeO_2_@HA, which could counteract bacterial imbalances caused by ROS and inflammatory factors. The predicted results of intestinal metabolic functions was obtained by using PICRUSt2 functionality. As shown in Fig. 7M, Res-CeO_2_@HA may be involved in sub-functions of pathways such as microbial metabolic pathways, biosynthesis of secondary metabolites, biosynthesis of amino acids, ABC transporters, and carbon metabolism at the level of Kyoto Encyclopedia of Genes and Genomes (KEGG) third-tier pathways. The findings indicated that the sub-functions of the pathways involved were generally consistent across all treatment groups. To explore the relationship between colitis and the gut microbiota, Spearman's correlation analysis was carried out to reflect the correlations between the microbiota and inflammatory cytokines, as well as enzymes associated with inflammation. These factors include IL-10, IL-1β, IL-6, TNF-α and MPO. In Fig. 7L, *Bacteroides*, *Acinetobacter*, *Odoribacter* and *norank_f__Muribaculaceae* exhibited negative correlations with IL-10 and had a positive correlation with IL-1β, IL-6, TNF-α, and MPO, which may contribute to the development of IBD. Studies have shown that *Bacteroides* is a harmful bacterium that promotes intestinal inflammation [87]. Dai et al. [88] showed that *Odoribacter* induces colitis. Conversely, *Blautia*, n*orank_f__norank_o__Clostridia_UCG-014*, *Alloprevotella* and *Lactobacillus* showed positive correlations with IL-10, indicating that these microbiotas may be beneficial in inhibiting the progression of IBD. Cheng et al. findings suggested that *Blautia producta* significantly alleviates DSS-induced intestinal inflammation by modulating ROS levels, suppressing excessive immune and inflammatory responses, and improving SCFA metabolism [89]. Mao et al. [90] showed that *Blautia* interventions significantly inhibited the transcription and expression levels of TLR4, MyD88 and caspase-3 in the liver [91]. Hence, these results illustrated that Res-CeO_2_@HA improved the structure and composition of the gut microbiota particularly by increasing the abundance of beneficial bacteria while reducing that of harmful bacteria.

## 4 Conclusion

In summary, a nanocomposite built from the resveratrol-embedded hollow cerium oxide and hyaluronic acid (Res-CeO_2_@HA) was developed as an effective strategy for the treatment of IBD. This nanomaterial enhanced the ROS scavenging capability of CeO_2_ while simultaneously enabling the targeted delivery of Res to inflammatory sites, result in improving its bioavailability in *vivo*. As the excellent ROS scavengers, Res-CeO_2_@HA effectively reduced the excessive accumulation of ROS and mitigated the secretion of pro-inflammatory cytokines in the colon, thereby inhibiting the development of inflammation. Meanwhile, Res-CeO_2_@HA promoted the polarization of M2 macrophages that released the anti-inflammatory factors in inflammatory microenvironment. And its protective effect on the intestinal barrier and the underlying mechanism may be related to the regulation of the TLR4/NF-κB signaling pathway. Furthermore, Res-CeO_2_@HA demonstrated a vital role in regulating the homeostasis of the intestinal microbiota, accompanied by an increase in beneficial bacteria as well as a decrease in harmful bacteria. Hence, Res-CeO_2_@HA could be regarded as a novel potential candidate for IBD treatment with the multifunctional in ROS clearance, inflammation treatment, intestinal barrier protection, and gut microbiota regulation.

## CRediT authorship contribution statement

**Tianlin Wang:** Writing – original draft, Resources, Methodology, Conceptualization. **Xiaoxia Lin:** Formal analysis, Data curation. **Wenjie Li:** Supervision, Software. **Xing Li:** Visualization, Validation. **Xiaodong Lin:** Project administration, Investigation. **Ning Li:** Supervision, Investigation. **Yan Ma:** Supervision, Software. **Lianjun Song:** Validation, Software. **Xianqing Huang:** Supervision, Investigation. **Tiange Li:** Writing – review & editing, Resources, Funding acquisition.

## Ethics approval and consent to participate

The experiments were received approval from the Animal Welfare and Ethics Committee of the Key Laboratory of Animal Immunology, Henan Academy of Agricultural Sciences.(approval number: LLSC41024036).

## Declaration of competing interest

The authors declare that they have no known competing financial interests or personal relationships that could have appeared to influence the work reported in this paper.

## Data Availability

Data will be made available on request.
