# Supplementary material for: Resveratrol-embedded hollow cerium oxide nanomedicine targeted treat inflammatory bowel disease through ROS clearance, intestinal mucosal immune homeostasis recovery and gut microbiota modulation
Source: Mater Today Bio. 2026 Jan 7;36:102765. doi: 10.1016/j.mtbio.2026.102765 (PMC12818119; doi:10.1016/j.mtbio.2026.102765)
Supplement: Multimedia component 1 [file mmc1.docx]

**Supporting Information**

**Resveratrol-embedded hollow cerium oxide nanomedicine targeted treat inflammatory bowel disease through ROS clearance, intestinal mucosal immune homeostasis recovery and gut microbiota modulation**

Tianlin Wang ^a^, Xiaoxia Lin ^a^, Wenjie Li ^b^, Xing Li ^b^, Xiaodong Lin ^c^, Ning Li ^a^, Yan Ma ^a^, Lianjun Song ^a^, Xianqing Huang ^a^, Tiange Li ^a*^

^a^ College of Food Science and Technology, Henan Agricultural University, Zhengzhou 450002, China.

^b^ College of Public Health, Zhengzhou University, Zhengzhou, 450000, China

^c^ Department of Bioengineering, University of California, Riverside, California 92521, United States

*Corresponding authors. E-mails: [litiange@heanu.edu.cn](mailto:litiange@heanu.edu.cn)


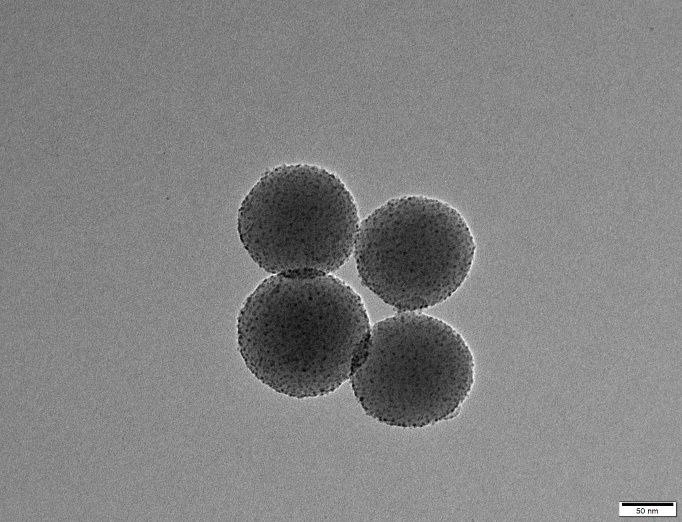


**Fig. S1** TEM images of SiO_2_, scale bar = 50 nm.


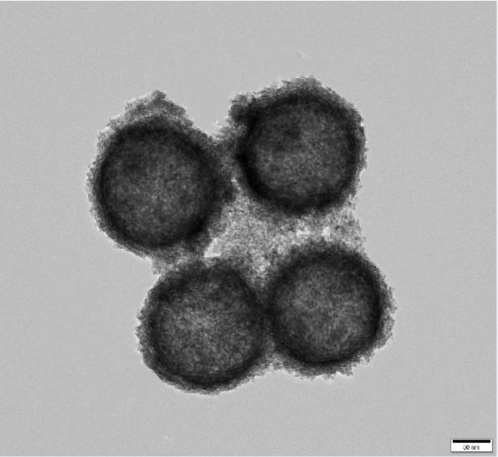


**Fig. S2** TEM image of CeO_2_, scale bar = 50 nm.


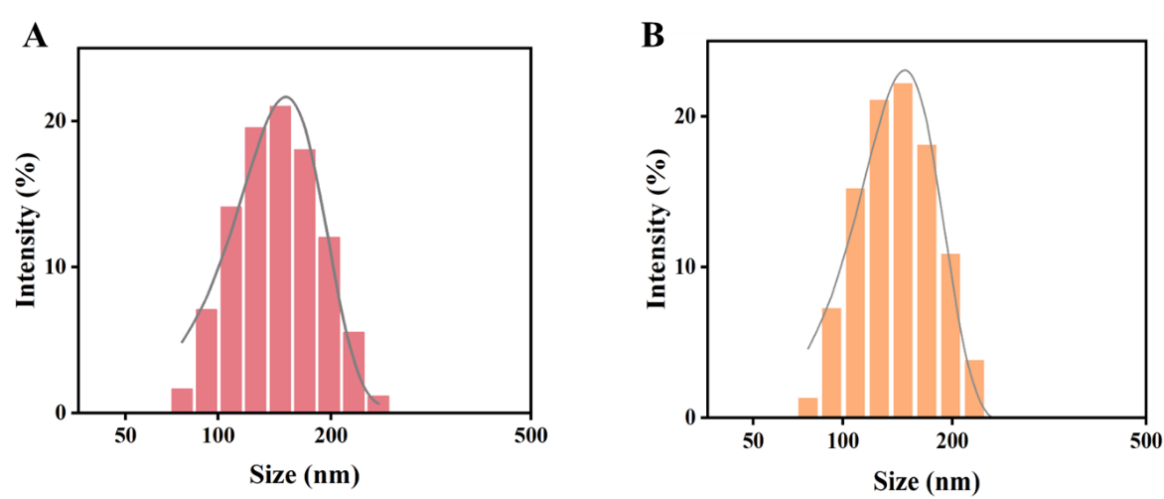


**Fig. S3** Particle size distribution of (A) CeO_2_ and (B) Res-CeO_2_.


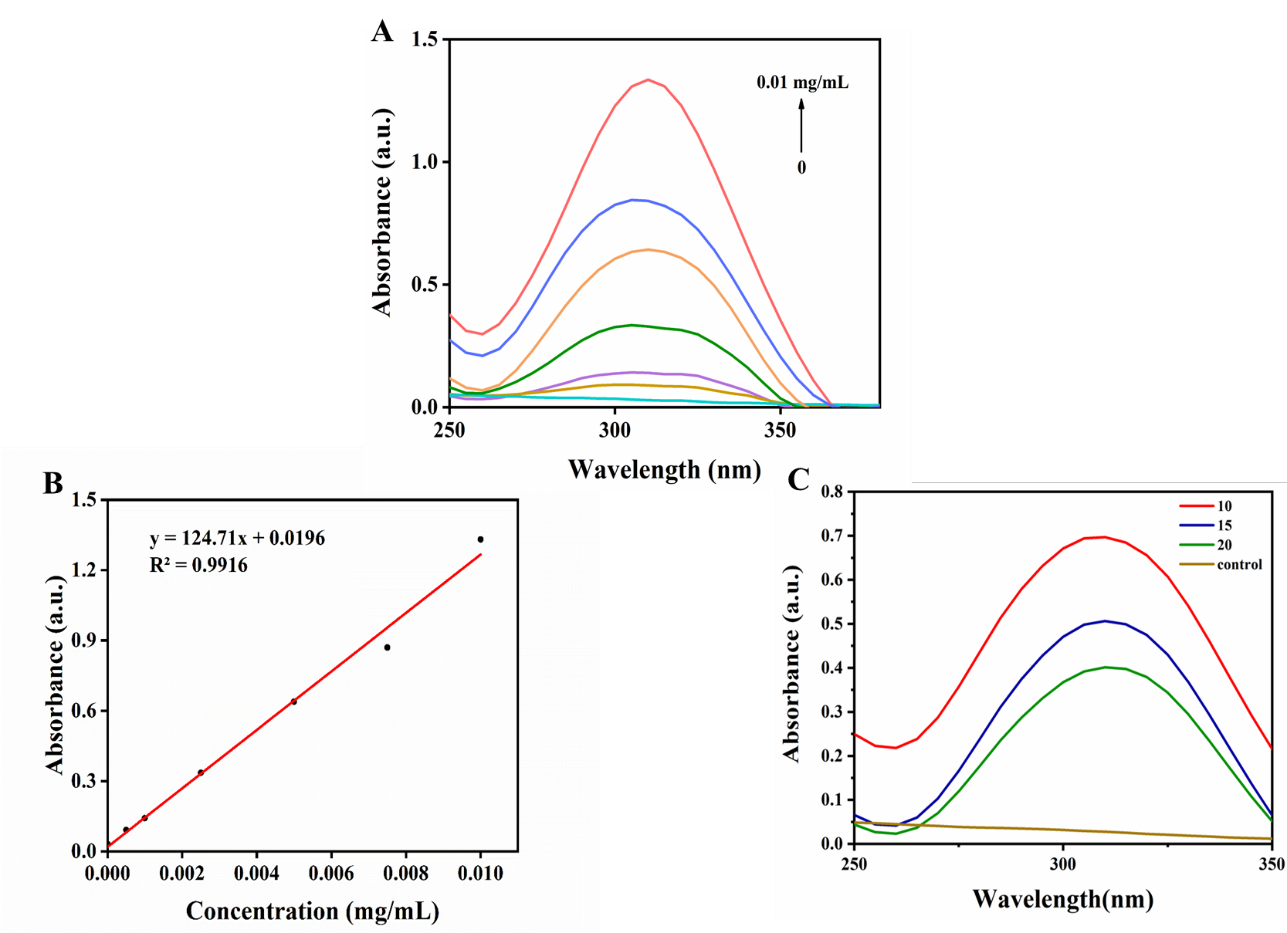


**Fig. S4** UV spectra (A) and Stander curve (B) of Res. (C) UV spectra of Res in the supernatant.


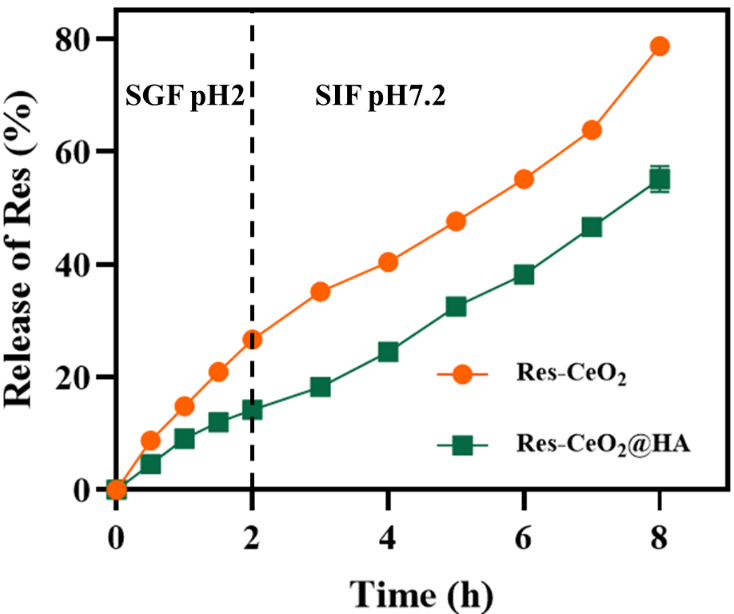


**Fig. S5** The release of Res from Res-CeO_2_ and Res-CeO_2_@HA *in vitro* under continuous simulated digestive fluid.


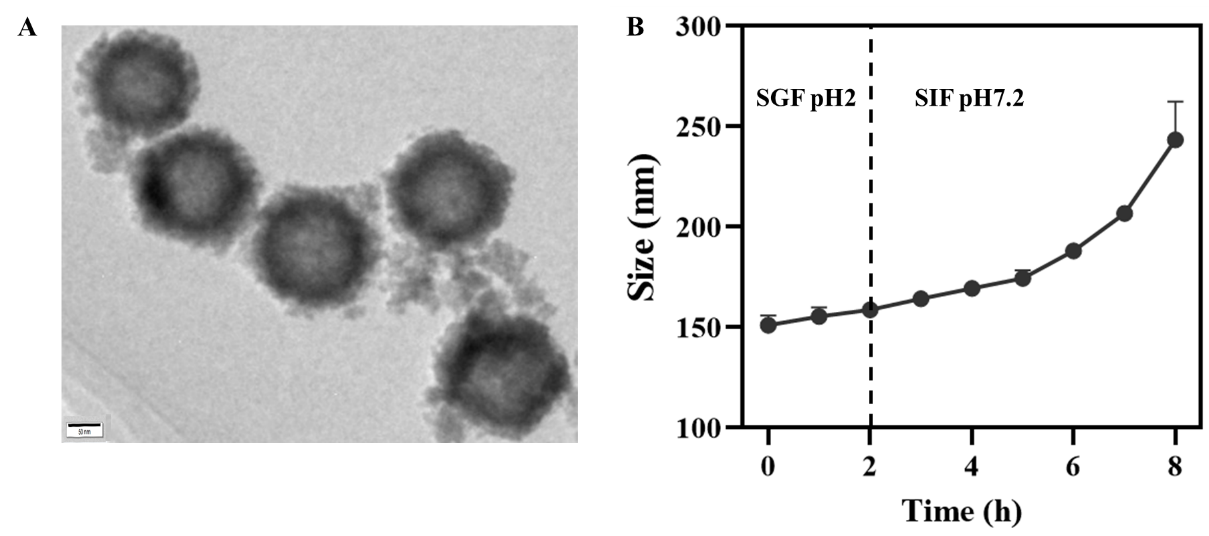


**Fig. S6** (A) TEM image of Res-CeO_2_@HA after 2 hours of simulated gastric digestion, scale bar = 50 nm; (B) Particle size change of Res-CeO_2_@HA during simulated digestion (n = 3)**.**


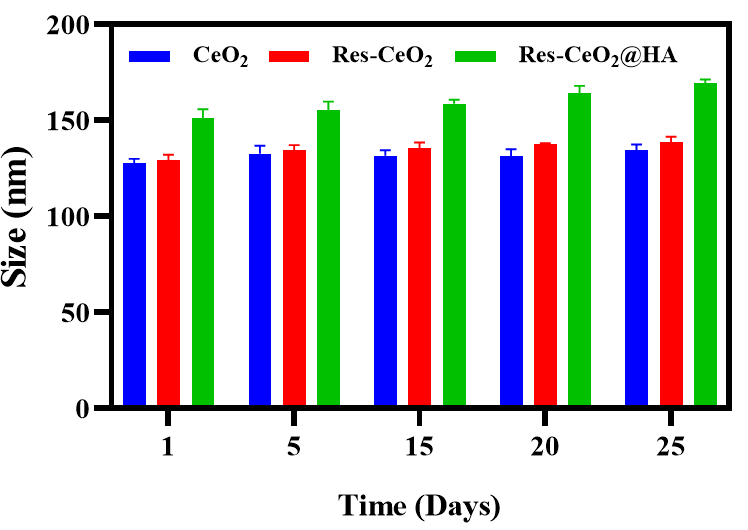


**Fig. S7** Changes in the particle size of CeO_2_, Res-CeO_2_, and Res-CeO_2_@HA at different storage times (n = 3).


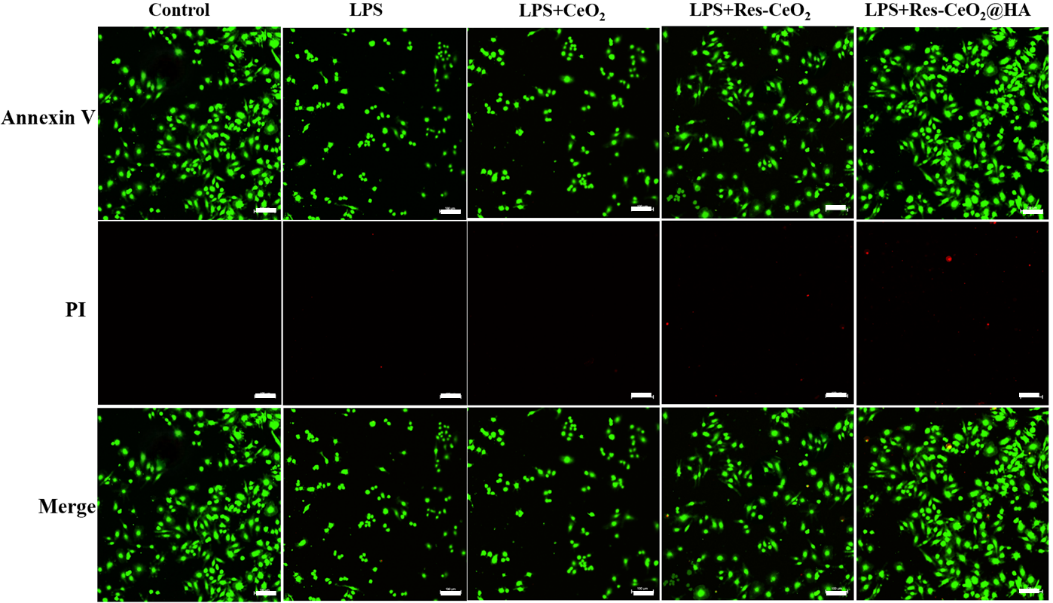


**Fig. S8** CLSM images of Annexin V-FITC/PI after treated with different groups, scale bar = 100 μm (n = 3).


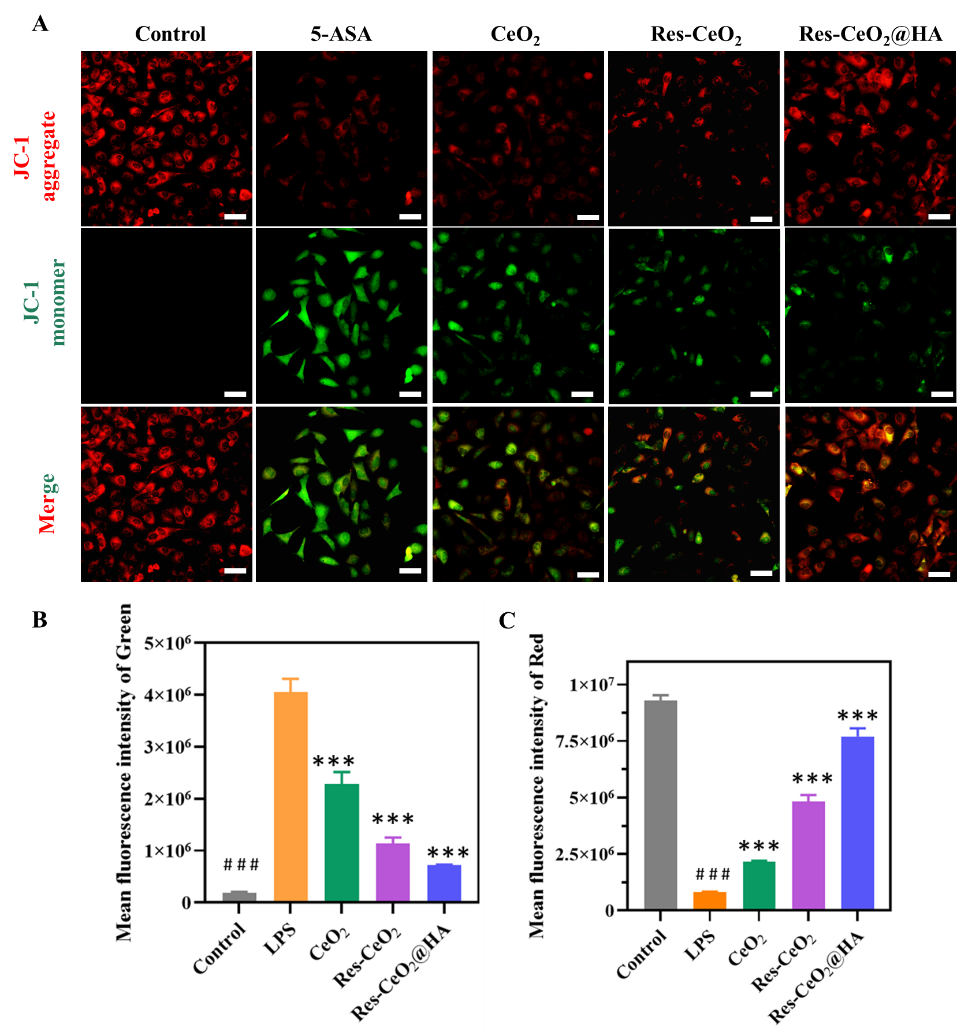


**Fig. S9** (A) The Δψm of mitochondria in different groups was investigated using confocal microscopy, scale bar = 50 μm. (B-C) Semi-quantitative results of the Δψm changes of mitochondria (n = 3).


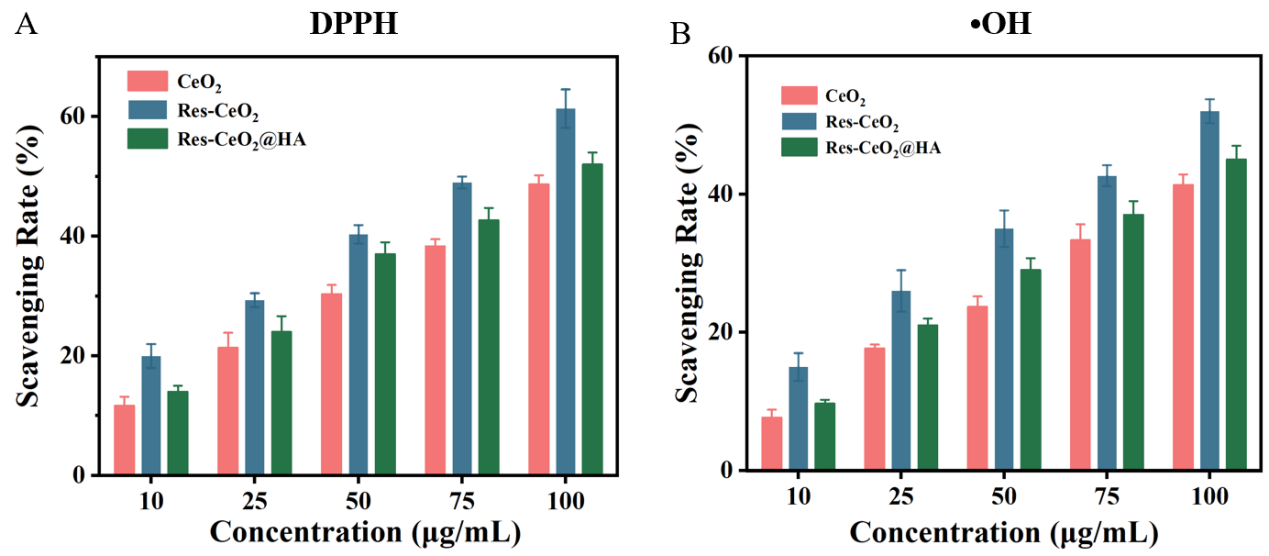


**Fig. S10** DPPH scavenging rates (A), •OH scavenging rates (B) of CeO_2_, Res-CeO_2_ and Res-CeO_2_@HA (n = 3)**.**


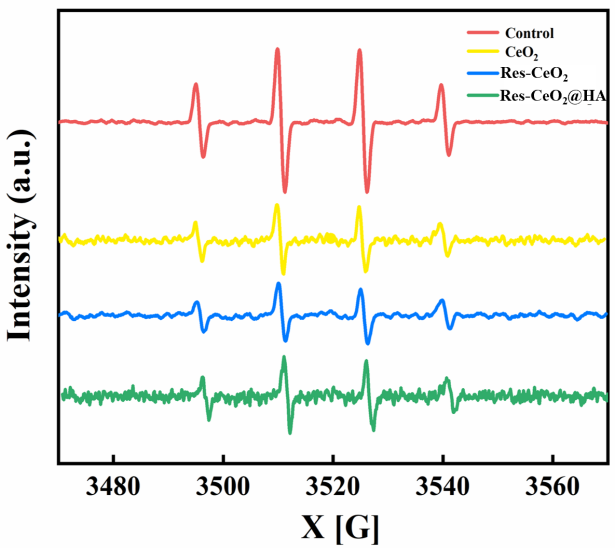


**Fig. S11** EPR spectra of CeO_2_, Res-CeO_2_ and Res-CeO_2_@HA.


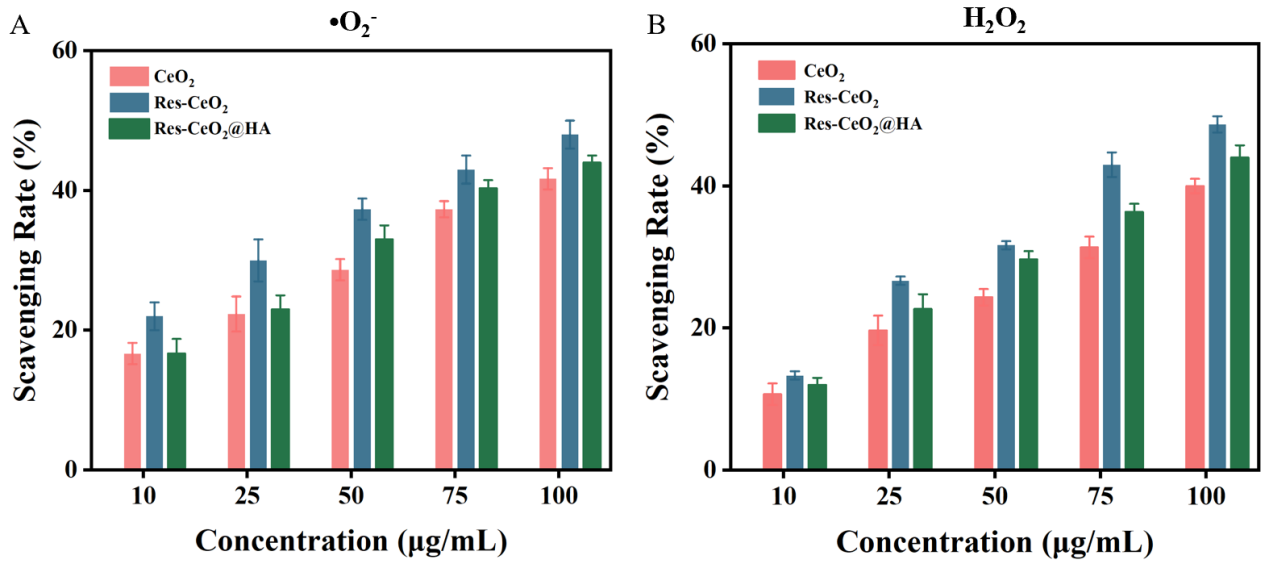


**Fig. S12** SOD-mimetic activities (A), CAT-mimetic activities (B) of CeO_2_, Res-CeO_2_ and Res-CeO_2_@HA (n = 3).


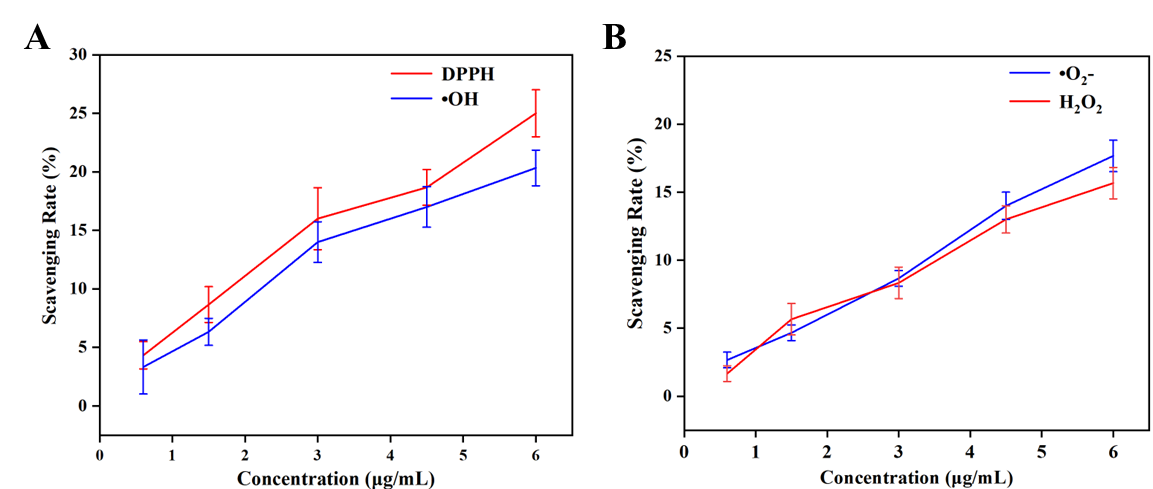


**Fig. S13** (A) DPPH scavenging rate and •OH scavenging rates of Res; (B) SOD-mimetic activities and CAT-mimetic activities Res (n = 3)**.**


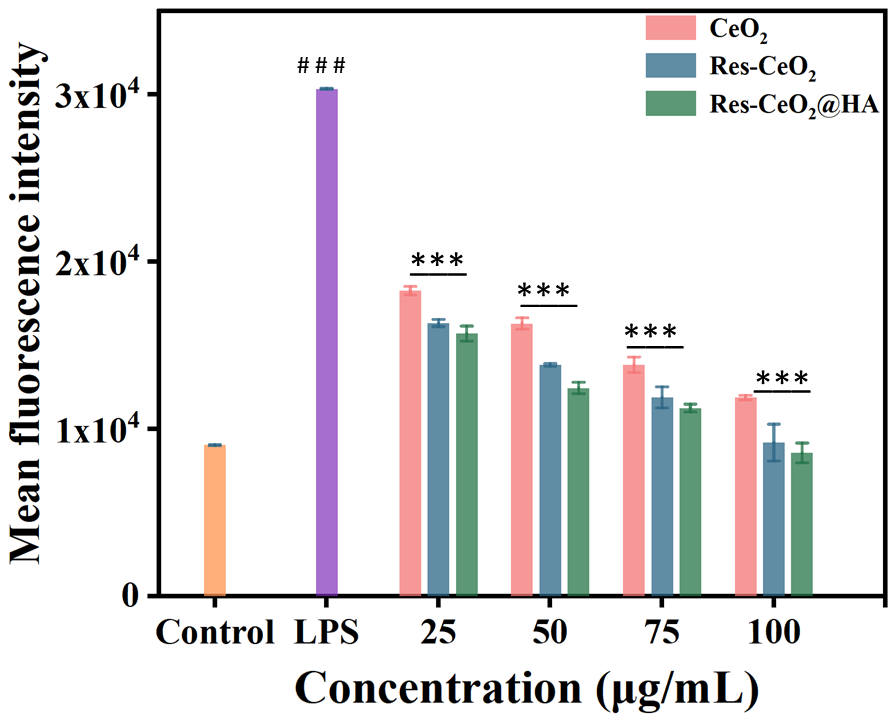


**Fig. S14** Fluorescent signal histogram indicating the ROS production in RAW264.7 cell after treated with different concentration of CeO_2_, Res-CeO_2_ and Res-CeO_2_@HA (n = 3).


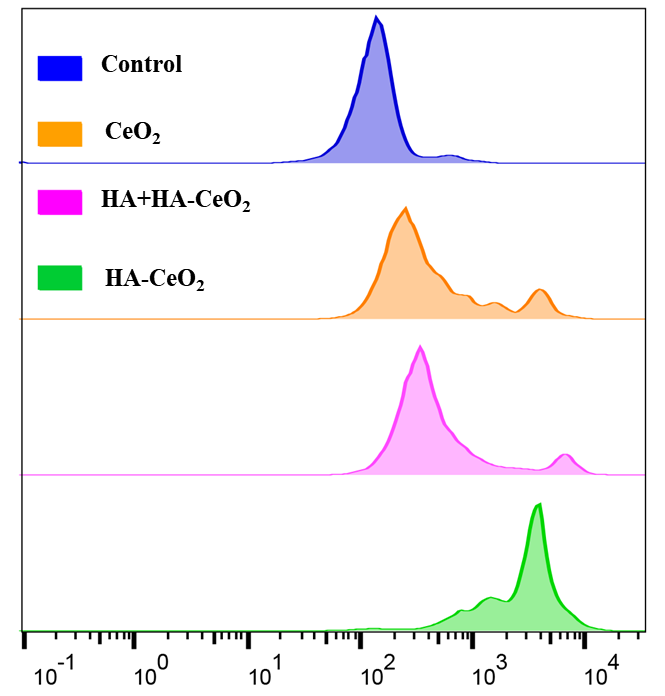


**Fig. S15** The flow cytometry of Cellular uptake.


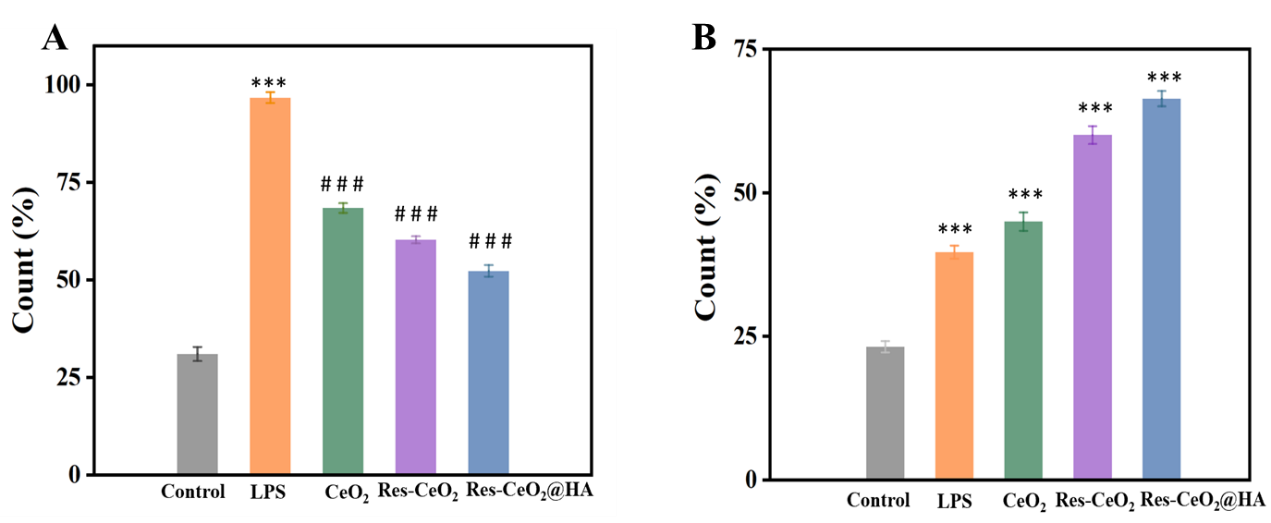


**Fig. S16** Quantification of (A) CD86-positive cells and (B) CD206-positive cells after treated with different groups (n = 3).


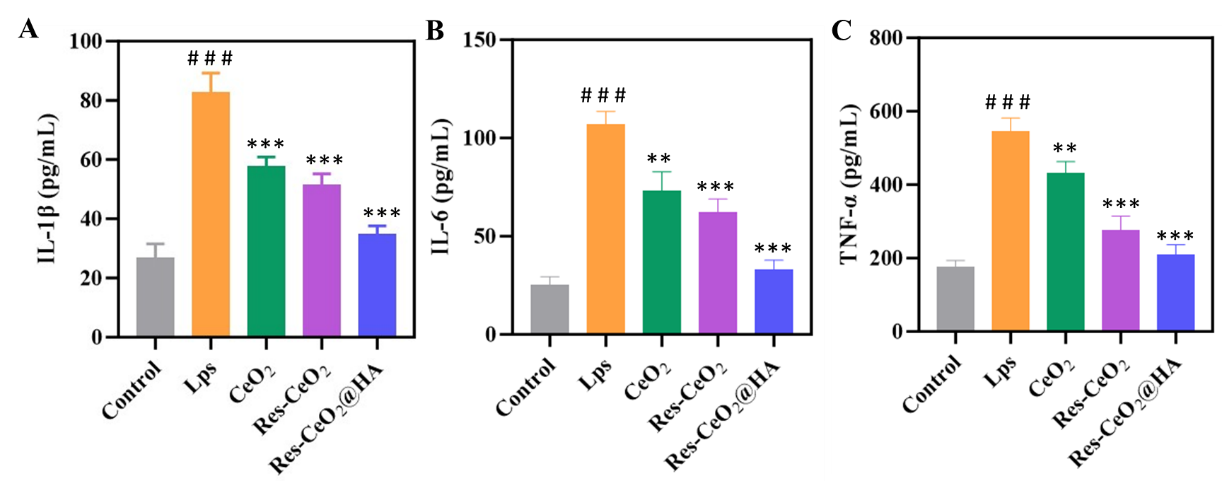


**Fig. S17** The levels of (A) IL-1β, (B) IL-6 and (C) TNF-α in cell supernatants of different treated groups (n = 3).


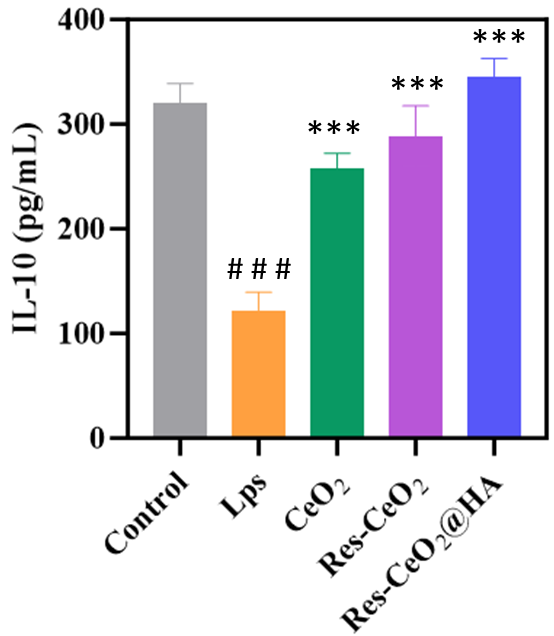


**Fig. S18** The levels of IL-10 in cell supernatants of different treated groups (n = 3).


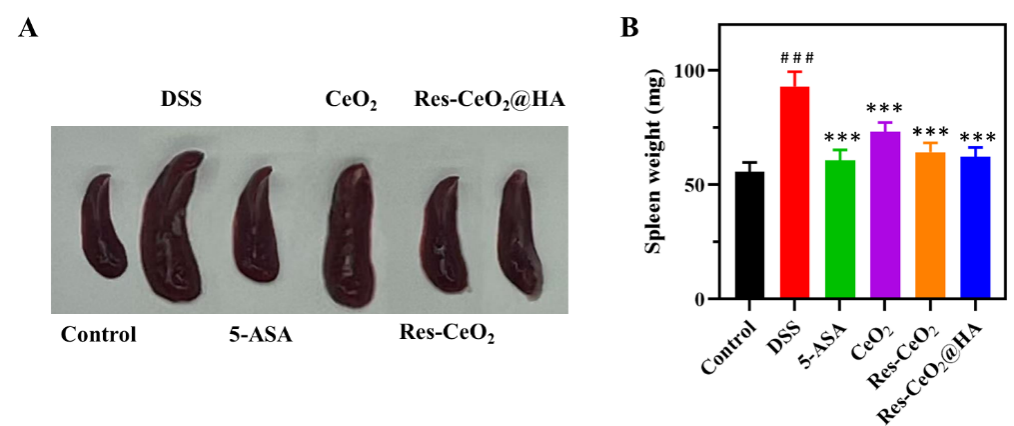


**Fig. S19** (A) Representative spleen diagram of mice in different group, (B) Quantification diagram of spleen weight of mice in different group (n = 3).


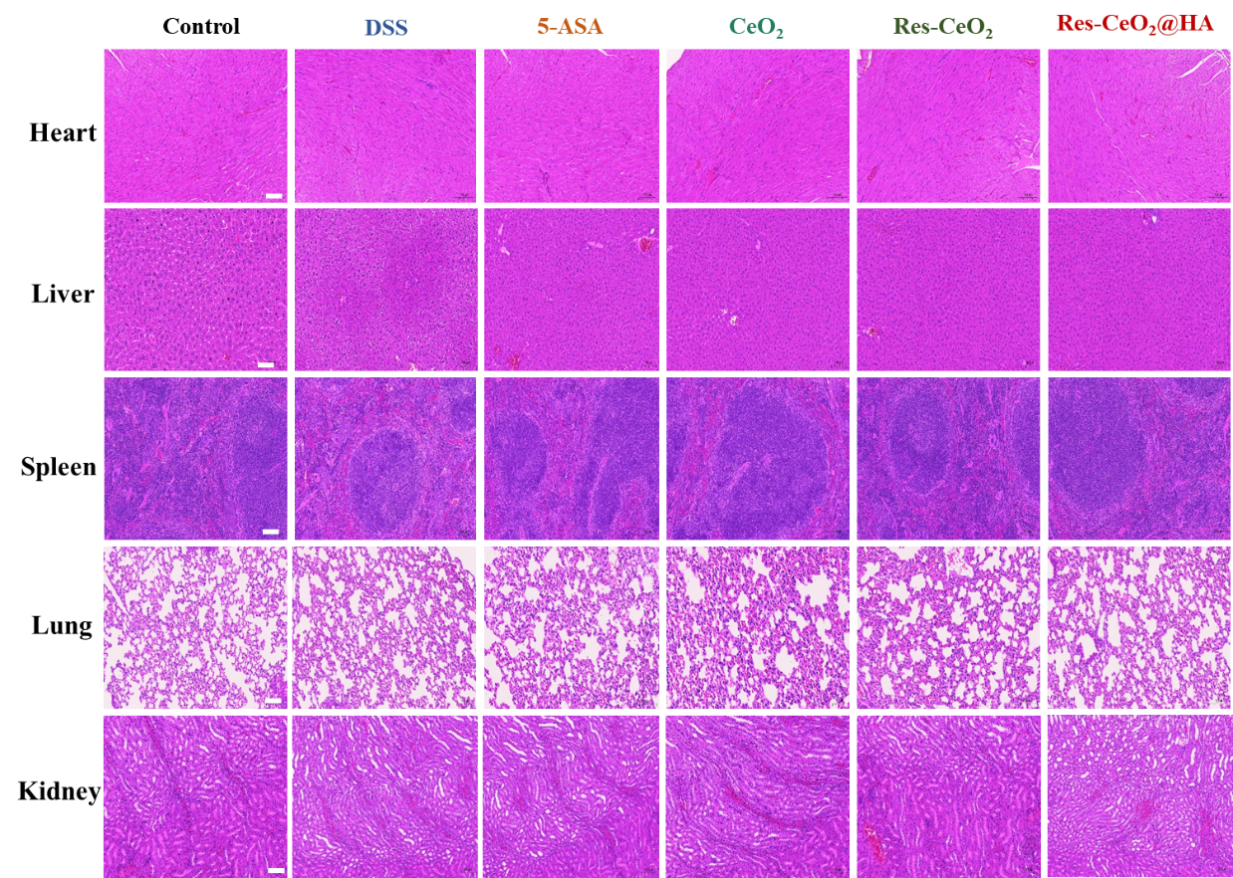


**Fig. S20** H&E staining of main organs (heart, liver, spleen, lung and kidney) in different treated groups, scale bar = 50 μm


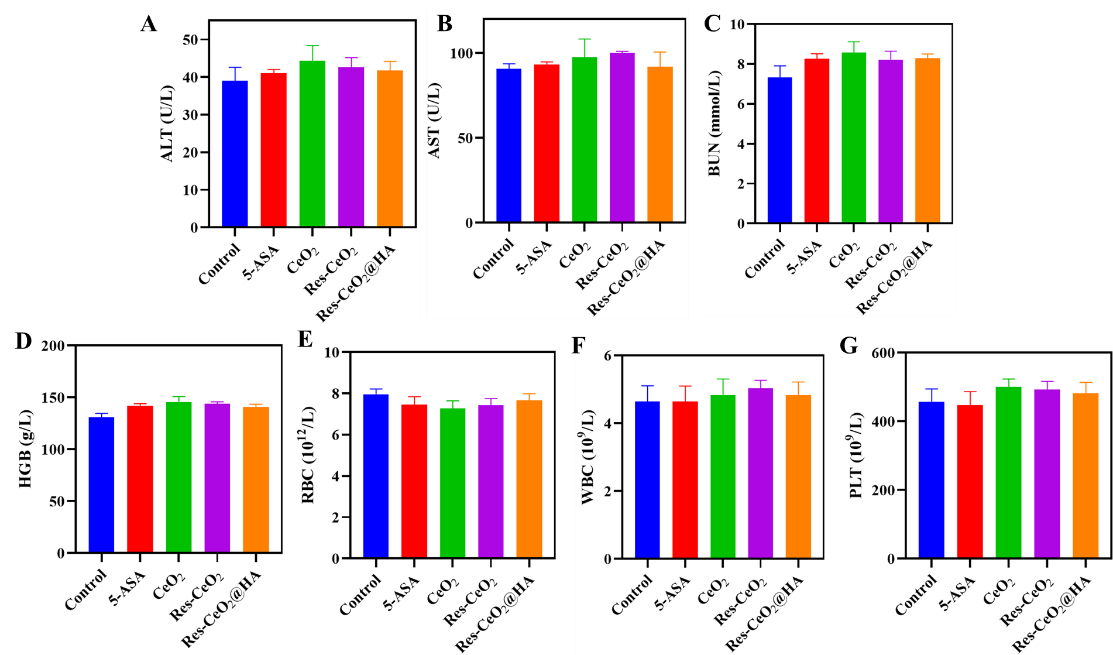


**Fig. S21** Serum levels of (A-B) liver function indicators, (C) kidney function indicators and blood parameters (D-G) in different group (n = 6).


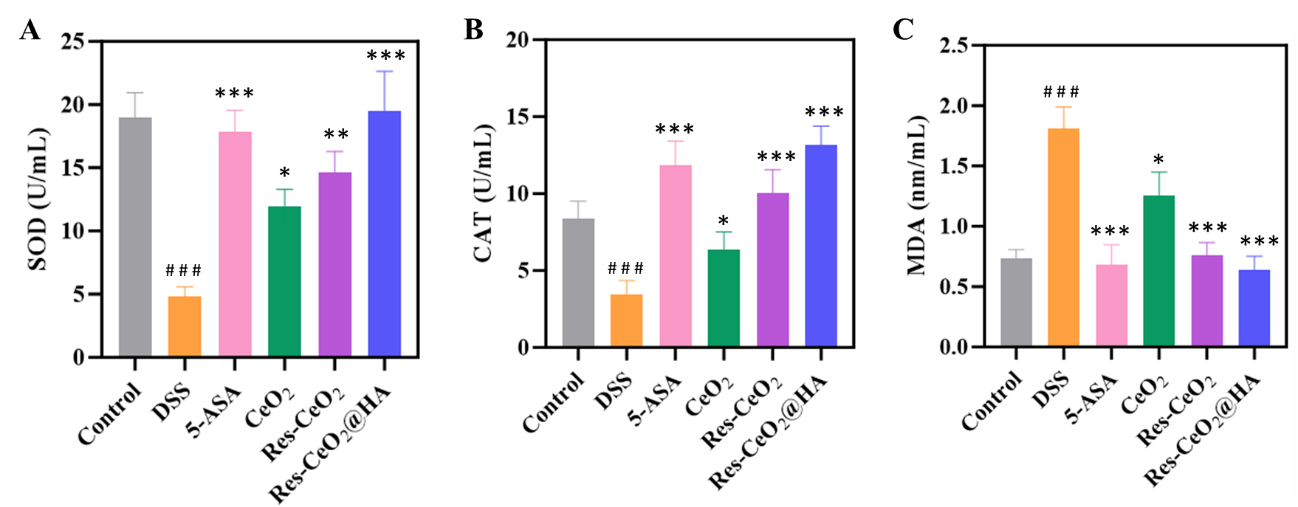


**Fig. S22** The levels of (A) SOD, (B) CAT and (C) MDA in serum of different treated groups (n = 6).


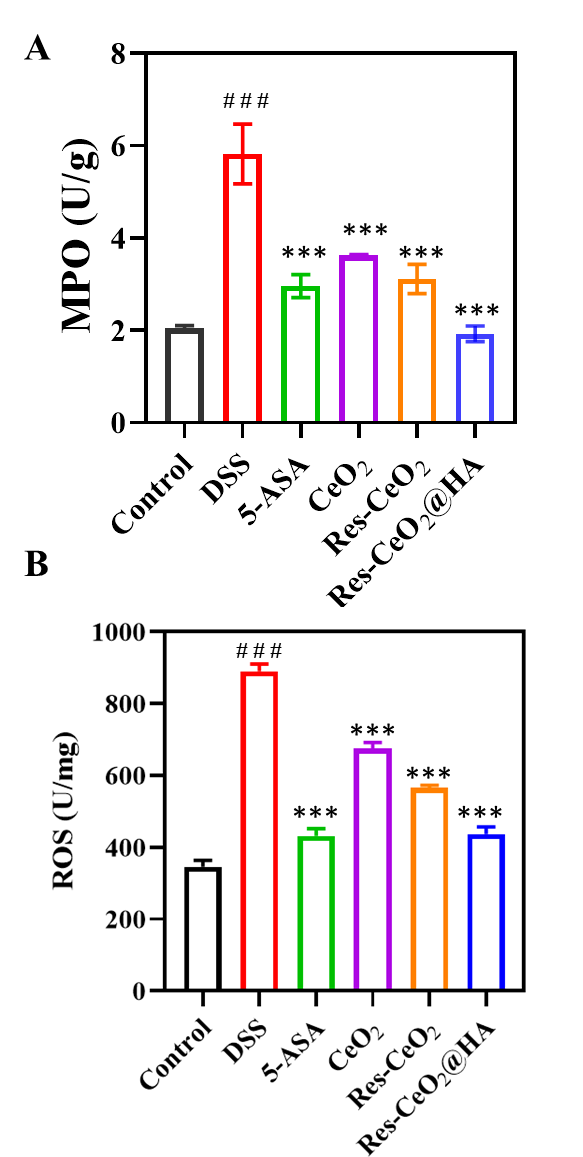


**Fig. S23** (A) MPO activity and (B) levels of ROS in different treated groups (n = 6).


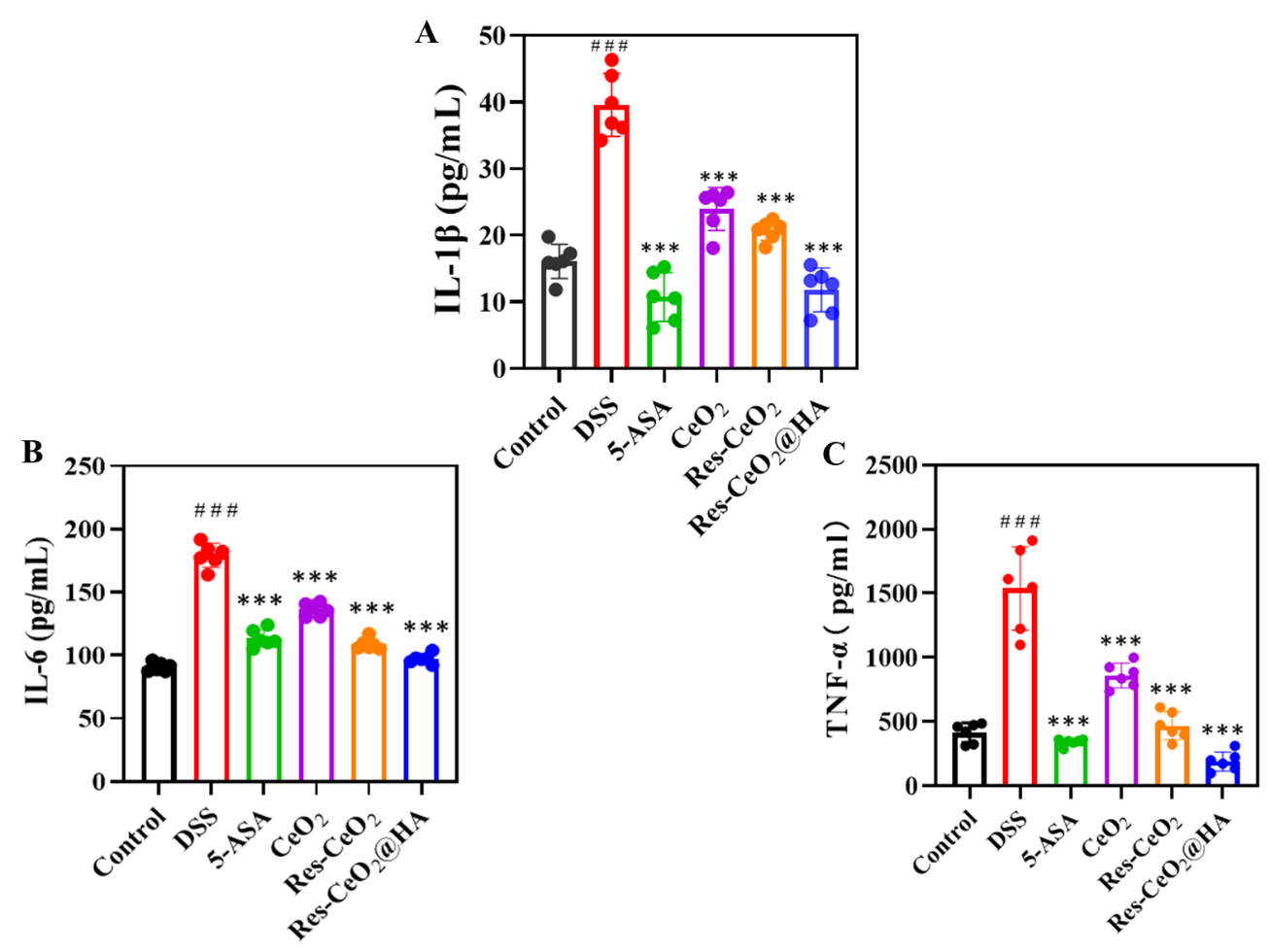


**Fig. S24** The level of (A) IL-1β, (B) IL-6 and (C) TNF-α in the serum (n = 6).


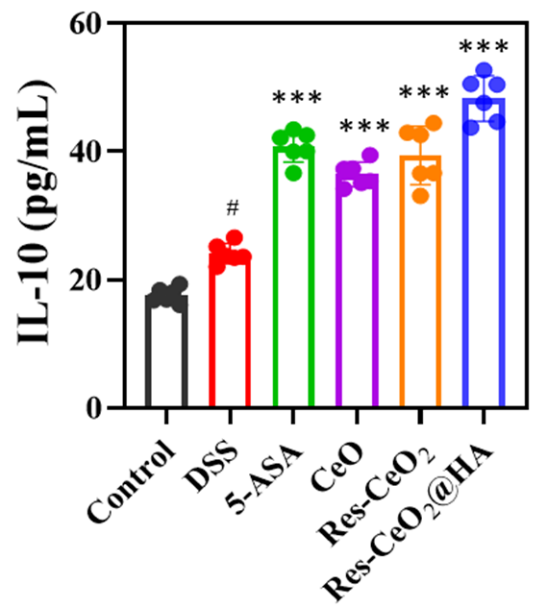


**Fig. S25** The level of IL-10 in the serum (n = 6).


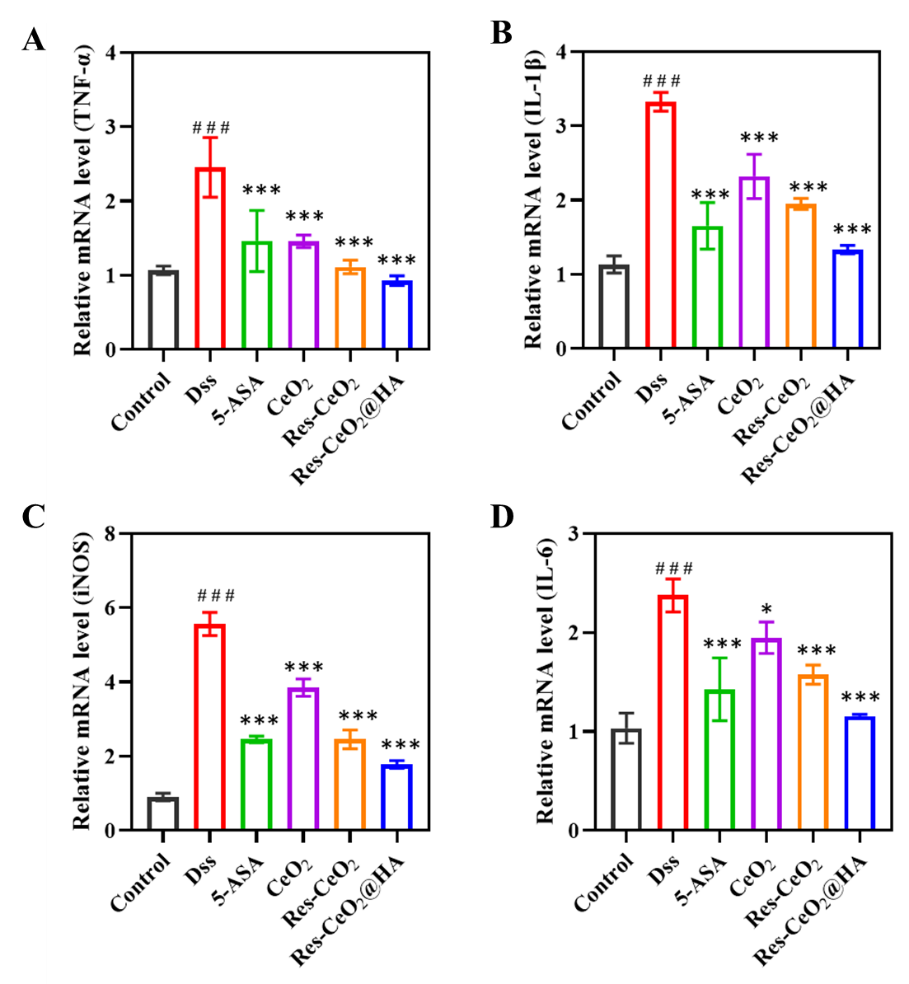


**Fig. S26** The mRNA expressions of (A) TNF-α, (B) IL-1β, (C) iNOS and (D) IL-6 in colon tissue (n = 3).


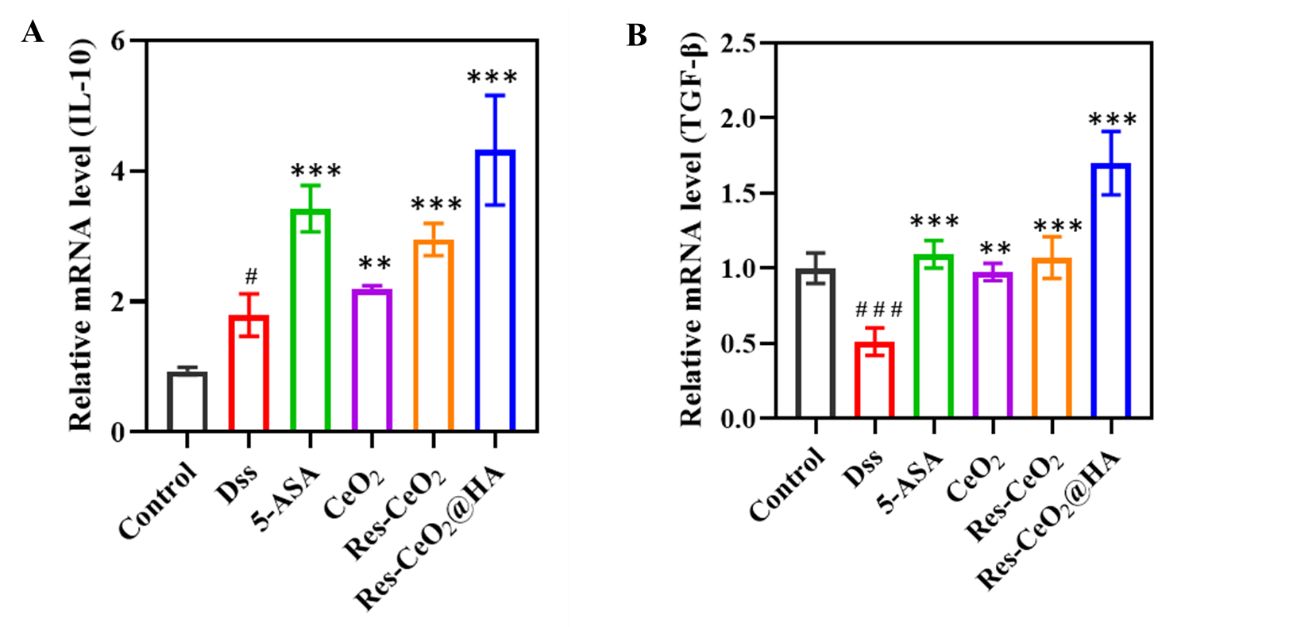


**Fig. S27** The mRNA expressions of (A) IL-10 and (B) TGF-β in colon tissue (n = 3).


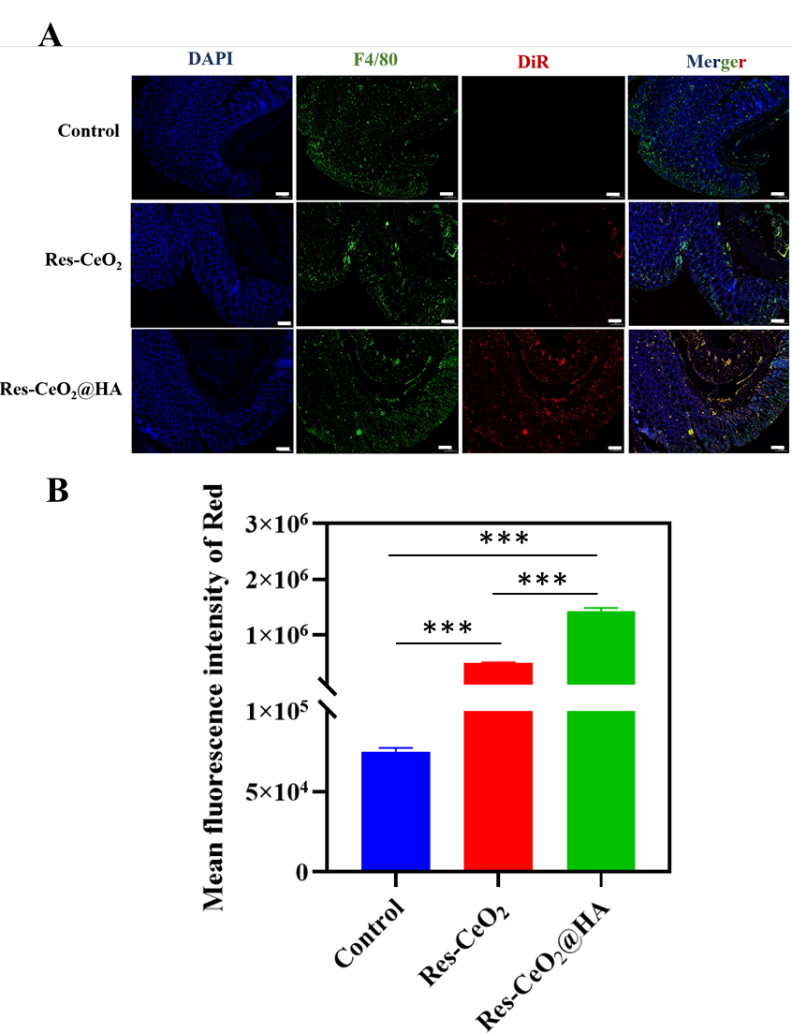


**Fig. S28** (A) Sections of colitis tissues after drug administration and (B) Quantitative analysis, Green: F4/80 Abs, Blue: DAPI, Red: DiR.


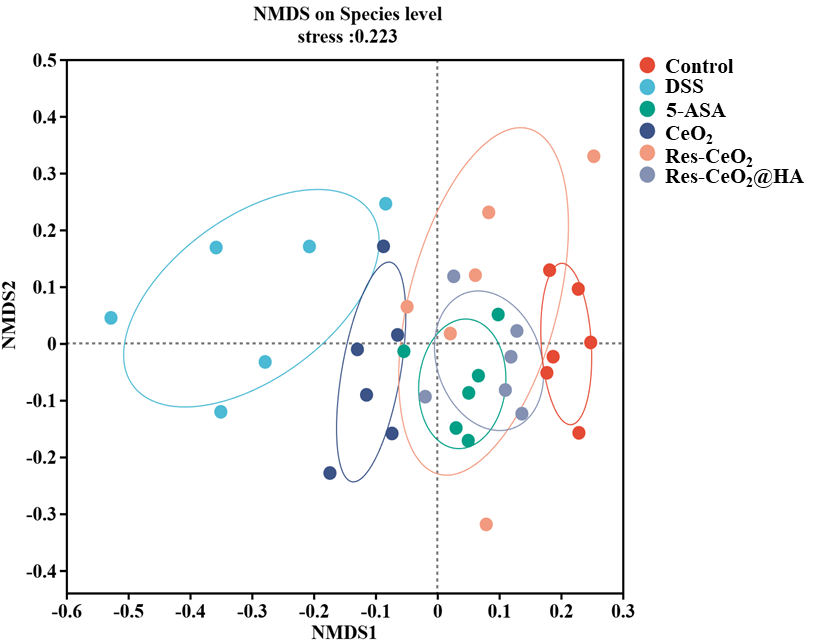


**Fig. S29** Non-metric multidimensional scaling on species level (n = 6).


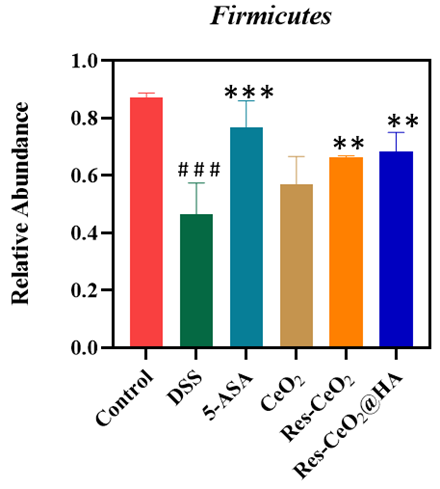


**Fig. S30** The relative abundance of *Firmicutes* at phylum level (n = 6).


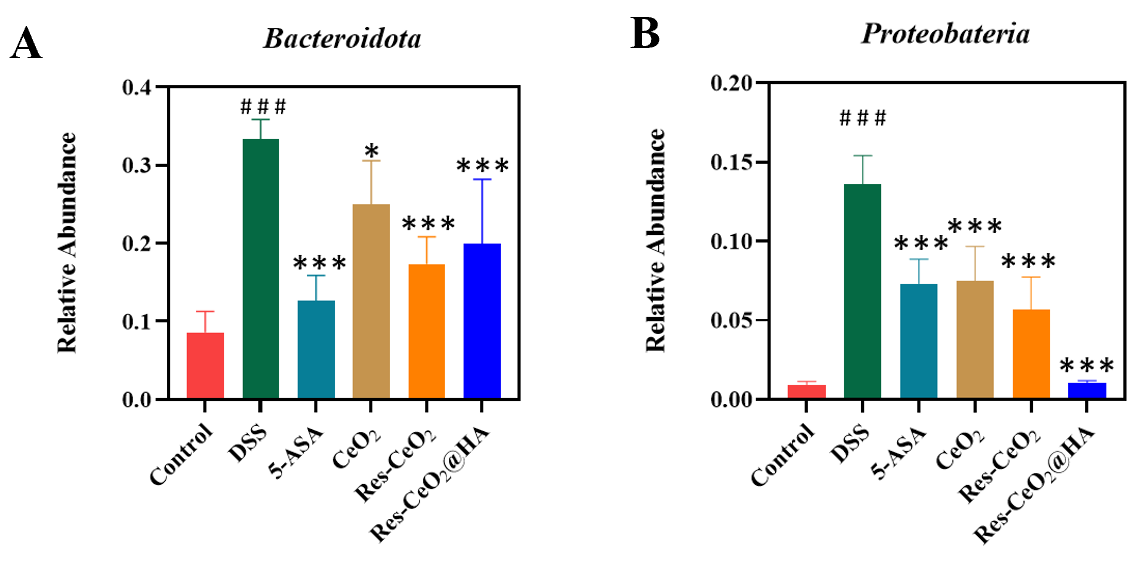


**Fig. S31** The relative abundance of (A) *Bacteroidota* and (B) *Protebateris* at phylum level (n = 6).


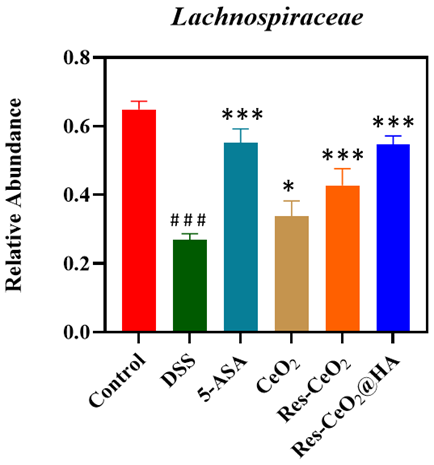


**Fig. S32** The relative abundance of *Lachnospiraceae* at family level (n = 6).


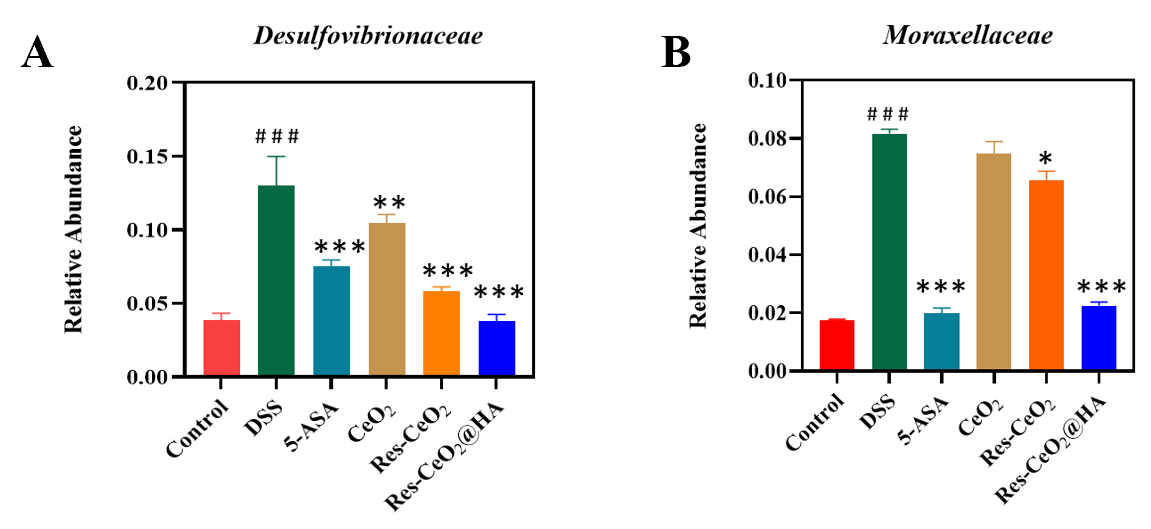


**Fig. S33** The relative abundance of (A) *Desulfovibrionaceae* and (B) *Moraxellaceae* at family level (n = 6).


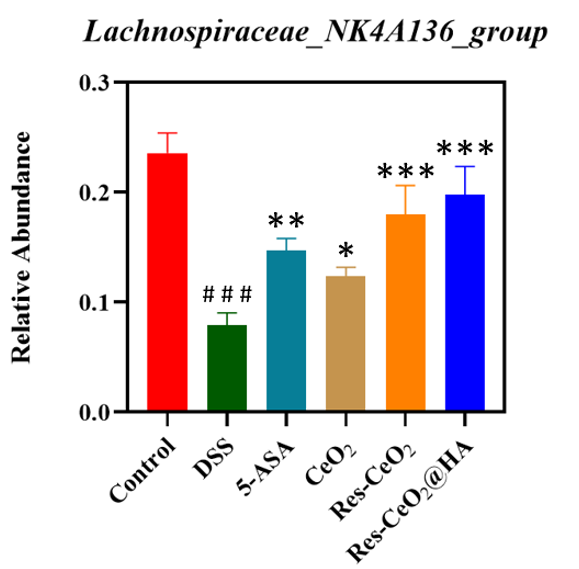


**Fig. S34** The relative abundance of *Lachnospiraceae_NK4A136_group*, and (B) *Desulfovibrio* at genus level (n = 6).


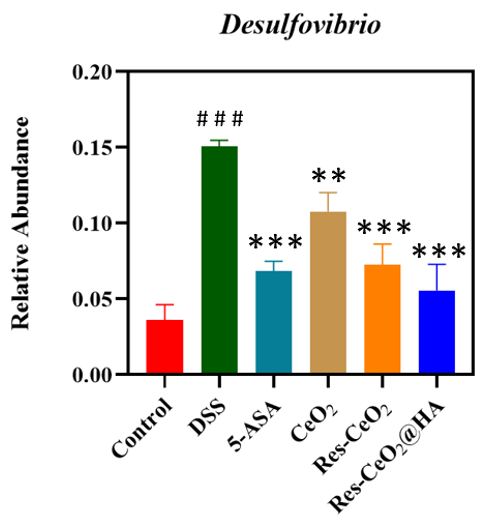


**Fig. S35** The relative abundance of *Desulfovibrio* at genus level (n = 6).

**Table S1** Primer Sequences

| Gene | Forward Sequence (5’-3’) | Reverse Sequence (5’-3) |
| --- | --- | --- |
| IL-6 | CCACTTCACAAGTCGGAGGCTTA | CCAGTTTGGTAGCATCCATCATTTC |
| TNF-α | CGATGGGTTGTACCTTGTCTAC | GAGGTTGACTTTCTCCTGGTATG |
| IL-1β | TCCAGGATGAGGACATGAGCAC | GAACGTCACACACCAGCAGGTTA |
| iNOS | CAGGAGGAGAGAGATCCGATTTA | GCATTAGCATGGAAGCAAAGA |
| TGF-β | CTTCAGCCTCCACAGAGAAGAACT | TGTGTCCAGGCTCCAAATATAG |
| Arg-1 | GCATTAGCATGGAAGCAAAGA | AGCAGGTAGCTGAAGGTCTC |
| IL-10 | ATGCTGCCTGCTCTTACTGACTG | CCCAAGTAACCCTTAAAGTCCTGC |
| GAPDH | ACCCTTAAGAGGGATGCTGC | CCCAATACGGCCAAATCCGT |

**Table S2** DAI Scoring Criteria

| DAI Scoring Criteria | | | | | |
| --- | --- | --- | --- | --- | --- |
| Stool Consistency | Hard | | Soft | | Diarrheal |
| Score | 0 | | 1 | | 3 |
| Fecal Blood | Negative | | Positive | | Grossly bloody |
| Score | 0 | | 1 | | 3 |
| Weight Loss | <1% | 1~5% | 5~10% | 10~20% | >20% |
| Score | 0 | 1 | 2 | 3 | 4 |
